# Supplementary material for: Fleshing out the data: when epidemiological researchers engage with patients and carers. Learning lessons from a patient involvement activity
Source: BMJ Open. 2020 Sep 30;10(9):e036311. doi: 10.1136/bmjopen-2019-036311 (PMC7528373; doi:10.1136/bmjopen-2019-036311)
Supplement: Supplementary data [file bmjopen-2019-036311supp001.pdf]

Appendix A: Topics of Dragon's Den tables (Feb 2017)

#	Table Topic	Research summary
1	Opportunities for reducing emergency cancer diagnosis: the role of comorbidities in influencing help-seeking for cancer symptoms and access to diagnostic investigations	We aim to examine the clinical events during the months before a cancer diagnosis in order to identify opportunities to diagnose cancer earlier and reduce emergency cancer diagnoses. In particular, we want to better understand why patients with a long-standing health problem (comorbidity) often have a higher risk of delayed cancer diagnosis and emergency presentation.
2	Examining inequalities in cancer survival for colon cancer patients living in London and treated with surgery in a London hospital	I am looking into identifying the main drivers of these differences in survival including factors such as: CCG of residence, hospital of surgery, socio-economic background, stage at diagnosis, specialised colorectal-surgeon, discussion of treatment plan in a MDT.
3	Evaluation of public health interventions	We aim to evaluate public health cancer policies soon after their implementation or even before their implementation. Using the technique of 'scenario modelling', by which we artificially change the composition of our cohorts of patients and how they are managed, to reflect the likely impact of a policy, we would like to look at the effect of those policies on cancer survival and other derived measures.
4	Factors influencing decision-making in cancer care.	These studies aim to explore which factors influence doctors' and patients' decision-making regarding the management of an aggressive type of Non-Hodgkin Lymphoma called Diffuse Large B-Cell Lymphoma (DLBCL) and/or bowel cancer. In particular, we are interested in people's attitude towards clinicians' management of elderly cancer patients.

**5**	Inequalities in cancer management and survival: NHS processes and/or lack of resources?	Some groups of cancer patients, for example from the more deprived categories, experienced lower survival from their cancer. It has been shown that these patients do not receive the most optimal management (including diagnostic investigation and treatment), and their higher proportion of comorbidity and more advanced stage at diagnosis would explain only part of these inequalities in cancer management. The reasons these patients are, for example, more likely to be managed in non-specialised hospitals or to be operated by less experienced surgeons are not understood. We aim to investigate the role of the organisation (processes) of the NHS and its disparities in resources, in these inequalities in cancer management and cancer survival.

Appendix B: Evaluation of relevant parts of the day given within a week of the event

	No new information	Reinforced existing information	Slightly informative	Informative	Extremely informative
Group session	0 (0.0%)	4 (13.8%)	8 (27.6%)	4 (13.8%)	13 (44.8%)
Plenary feedback session	0 (0.0%)	4 (13.8%)	6 (20.7%)	6 (20.7%)	13 (44.8%)
	**Topic not relevant to me**	**Relevant but not useful**	**Slightly useful**	**Useful**	**Extremely Useful**
Group session	1 (3.4%)	6 (20.7%)	5 (17.2%)	6 (20.7%)	10 (34.5%)
Plenary feedback session	1 (3.4%)	4 (13.8%)	7 (24.1%)	7 (24.1%)	9 (31.0%)
	**Ineffective**	**Neither effective nor ineffective**	**Slightly effective**	**Very effective**	**Exceptional**
Table hosting	2 (6.9%)	1 (3.4%)	13 (44.8%)	9 (31.0%)	4 (13.8%)
	**Unsatisfactory**	**Neither satisfactory or unsatisfactory**	**Slightly Satisfactory**	**Very satisfactory**	**Exceptional**
Event organisation	0 (0.0%)	1 (3.4%)	1 (3.4%)	21 (72.4%)	6 (20.7%)
Building and location	0 (0.0%)	0 (0.0%)	2 (6.9%)	20 (69.0%)	6 (20.7%)
Room and comfort	1 (3.4%)	2 (6.9%)	9 (31.0%)	12 (41.4%)	5 (17.2%)

Appendix C: Semi-structured interview questions

Interview questions for consumers

1. What were you hoping to gain from participating in the Dragon's Den session?
2. Did it meet your expectations?
3. Do you feel your comments were listened to and taken on board?
    a. How did the researcher make that possible?
    OR b. What was it about the session that meant that didn't happen?
4. Did you feel that the feedback provided by the consumers will make a positive difference to the project?
5. Did you feel that there was enough opportunity for questions and discussion?
6. Was the session the right length/layout/number of participants?
7. Do you have any comments or suggestions for improvements that we could make to the session in order to better meet the needs of researchers and/or consumers?
8. Any other comments?

Interview questions for researchers

1. What were you hoping to gain from presenting at the Dragon's Den session?
2. Were you able to achieve those objectives?
3. Did the session meet your expectations?
4. Do you think the feedback provided by the consumers will make a positive difference to the project?
    a. In what way?
    b. Or why not?
5. What do you think the consumers got out of the session?
6. Did you feel that there was enough opportunity for questions and discussion?
7. Was the session the right length/layout/number of participants?
8. Do you have any comments or suggestions for improvements that we could make to the session in order to better meet the needs of researchers and/or consumers?
9. Any other comments?

Appendix D

Box 1

Overall experience (what did you get out of the session?)

It's an opportunity to hear what other people think about something, expanding my own learning and it gives me more experience that I can draw on in the future…So for me it's about trying to get my bit across, listen to everyone else, and try to learn a bit more, it's about a learning process (C2)

All the topics were relevant, it was well planned, relevant to patients with cancer, living with and beyond cancer… There is a feeling of altruism wanting to give back and help someone's knowledge and experience so perhaps this information will help others being treated in the future…I would like to see it repeated because I think it was a very fruitful event. (C3)

I enjoyed it very much and got a lot out of it. (C4)

I was thrilled at that opportunity to discuss with Consumers...it was a great experience, and I wish we had more opportunities to share about the work we do in that context (R1a)

I thought it was a very useful exercise and patient representatives were really active and each of them really had something to say on the topic. So, I think it was a really useful interaction with patients. (R3a)

It was the first time I was involved in the Dragons' Den, I would like to definitely to repeat it (R4a)

I was excited to get some information from people with first-hand experience of cancer. (R4b)

On the actual day, the sessions were largely as I had expected them to be. The participants were very…keen to share their views and experiences, which were very insightful. (R5b)

It was a lot more difficult than I thought. I didn't really know what we were there for, so it was a bit challenging at times. (C1)

I was also aware that there may not be easy answers to our research question. The session was interesting but also quite difficult. (R4b)

Because it went so well actually I wanted to go beyond to have more information, to discuss more etc, and that was quite frustrating. And for everyone I think. (R2a)

I was a bit stressed, to start with, because I had no clue…if I would have to do the talking non-stop or the listening non-stop, and I think it went well overall, but it kind of got out of control…so in the end, I was happy when it finished, it was enough. (R1a)

Box 2

Expectations / Importance of patient contribution

Working together towards improving outcomes for patients is the best way to achieve reliable results… the genuine information can come from those who have experienced the situation undergoing that, dealing with things…As a colleague on an equal basis everyone working side-by-side to discuss the research and receiving equal consideration it's so important I think to provide this opportunity. (C3)

I believe the answers provided by the Consumers will provide more clarity on the question and the practicalities to be considered in the research. (C4)

You don't always know whether you are really being listened to or not because people are polite generally speaking, so therefore they sometimes can maybe appear to be interested. You don't know when they go away, whether they actually do anything with it or not. But I was aware in this case that they did, so that was very positive…I mean the researchers have a very good idea of the overview of what they want to get to, but sometimes it's the practical issues that the PPI [Public and Patient Involvement] can throw in. (C5)

It obviously did turn out to be pertinent. They showed that by their response at the time and I thought that those issues were incorporated in the grant proposal that we got to read later on…So, it was actually made very obvious that they did take that on board… (C5)

I don't know that I've heard very much what about what will actually happen, what the next steps are…it might be useful if there's something you could pass around at the end of the session to say "This is where this particular project is, here's the written summary of what you said" just some way of feeding that back. It was all said verbally. (C2)

So, I was hoping to get somehow, an insider's view of what matters to the patients really in terms of, or what they think could improve survival, somehow, stuff that could be addressed by policy makers. (R1a)

I think that it's useful to have the views from the patients to know that you're on the right track…And I hope they see that we value their point of view and their opinion and their contribution and how we have the need to hear what they think about the issues we are trying to study. (R3a)

The patients were well informed; their answers were precise, informative, and not just anecdotal. (R2b)

It brings a different perspective and different insights. (R4a)

It was touching to listening to their experiences and I appreciated the fact that most people were open about their personal experiences. (R1b)

Like all these things, they seem very obvious to them, but of course you won't get that sense from looking at registry data... It was a good reminder for us that we sit in front of the computer and it loses that personal touch…it felt really intense to hear their experience. (R5a)

Box 3

Content of discussion

There were a lot of us that for a while didn't really understand what was being asked of us and how we could help with the research. We did eventually but there was quite a while where we weren't quite sure for a while what exactly we were supposed to be inputting…it took us a while to figure out what the point to it was exactly. That wasn't clear at all, it was the nature of the study I guess (C1)

I think at the beginning of the session, it wasn't as clear as it could be exactly what the researcher was wanting to ask us. (C4)

There were sort of two bits to it, so it would have been easier if there had only been one piece rather than two angles. (C5)

There were a few steps that the Consumers needed to understand before they could really answer things that would be useful to me…So all of these concepts were quite tricky to grasp, and I discovered it as we were talking with the patients as well…It's more of a stats [project] so for me it was hard to translate into words and applications, and for them it was hard to get the stats concepts behind…I would have picked another subject I guess. (R1a)

I presented a very broad project…but I think it was too broad. (R4a)

But after a bit of fumbling we were definitely able to come up with a lot of stuff (C1)

We didn't have enough time to discuss: "So what? What can we do etc?" What I wanted to discuss, "Now there are some initiatives to try tackle that, or maybe not, we don't know - what do you think? Do you think it worsens the situation or does it help?" And we didn't have time and I think it would have been very useful. (R2a)

Box 4

Composition of groups

The number of participants, I think it was a good number and it was useful to have to people from different experiences, ages, and carers and patients and so I think it was a good mix and the number was right... And the fact that they were men and women, different age groups, I think that was very interesting, carers and patient[s]…If there was a bit more variety in terms of background of the participants, it would maybe make the discussion even richer, even open up new insights…The only downside I think was that these were all people who were highly engaged, eloquent, had very clear views, had thought about it a lot. So, when we

very briefly talked about the issue of lower SES, obviously there wasn't anybody there representing those groups so that was a bit of a limitation. (R3a)

I thought the number of participants was good, I thought the breadth of experience of the participants was good… In terms of representation, that was very good (C4)

I think it was fine. I wouldn't put more. But less is maybe too - you may not achieve what you want. I think you need the diversity, but not too many people. (R2a)

I think less people, not many less people but a few less people around the table would have been good, especially for the length of time… (C1)

…we are not representative of the population. It's the same with most patient reps - it tends to be…well educated white middle class with sharp elbows…they have resources in terms of time and education, can put some time aside and can make a difference, but we're not necessarily reflecting the population that we should reflect, given that cancer is a disease, certain cancers are more prevalent within certain communities. (C2)

I went away with the feeling that this is such a selected group … they are active patients because they're involved in these sort of things... Because they are there, and they are patients who they know that they can be useful for research (R5a)

Box 5

Facilitation of discussion

I thought [the researcher] was an excellent facilitator. Fortunately, Consumers are very talkative, so it's quite a job to not just comment and keep things going but try and keep things within the thing that you're supposed to be talking, trying to keep things moving along and I think that was done well (C2)

Yes – it was an open discussion, and everyone at the table could contribute and those that weren't able to easily contribute for example, having hearing difficulties, everyone was made to feel that had the same input as others, in a position where they were able to contribute appropriately…, I do remember the researcher being engaged with everybody. (C4)

[The lead researcher] did a good job in describing the topic and engaging members and handled the questions well by triggering different conversations (R1b)

I think it was tricky as the presenter to be presenting my topic and also moderating the discussion because I don't have those skills, of a moderator, and I also had to get my point across and I had to welcome everyone's suggestions so that everyone would feel free to discuss and that was quite tricky…It was really good that I didn't have to take the notes, because I think that would have been really hard… I made sure I was engaged in making eye contact. (R1a)

I think if maybe it had been a more senior person there … who is almost monitoring keeping an eye on what's going on, giving a helping hand if somebody looks a little bit adrift, they could intervene at that point and get things back on track for them… if you can use a whiteboard or a flipchart, not to make it too long, but a picture always can speak a thousand words and if you can draw a little diagram or a couple of words that sound very difficult, write them up so that people are clear what you're talking about. It makes it a little bit easier both for them to explain and for the other people to understand (C5)

[A consumer] was the one that actually tried to somehow focus the others, saying, "No, wait a minute, we're here to discuss this and this," because obviously [we were] not reaching that common point of understanding for all…I think the key is the structuring a priori because that would avoid … cross talking because it's natural, it's open and that's what happens (R4a).

Box 6

Layout of the room

The acoustics weren't very good, and the layout of the table was such that sometimes it was hard to hear what was being said by maybe another discussant at the far end...Either the room needed to be a little bit

bigger, so that the tables were more spaced out, which is what we would generally have. Or needed to be in a separate little sub room where you could talk a little bit more readily. The main thing I felt really was that the room that was used wasn't terribly great, it was squashed and too noisy and that caused issues (C5)

…maybe separate rooms with complete silence around would have helped a little bit. (R1a)

…there were loads of tables in the one room. It was actually quite difficult to hear so that was frustrating in some ways because on that small table if there had been more space a lot of us would have got a lot more out of that particular meeting (C2)

…it's always that thing of hearing what's being said at other tables, adjacent tables, but that's really environmental and beyond the control of the school itself. I do wonder if other participants…might feel a little hesitant about expressing, describing their personal situation…(C3)

For me I'm a little deaf, then it was hard because we had a lot of tables, it was a little noisy...we would need probably more space because the acoustics are not really good…You know, in a way it's nice to see this, I think it's - you know you are part of a big event in a way. It can be quite nice. While if you are all in different rooms… (R2a)

The room was loud, and a participant complained they could not hear or contribute to our discussion. (R4b)

Box 7
Consideration of special needs

There were a couple of people at our table who were a little older. And because [of that] they weren't as quick to pick up in terms of hearing, and they found the noisy background a bit of a distraction at times. (C5)

I think the noise could be a problem for anyone with hearing difficulties…there was always a low buzz of conversation which is always natural when people are speaking (C3)

…because there were a couple of people at the tables who had hearing issues, and they did find it hard to hear, so that could have been done better. (C4)

It was quite a full-on day, all day long and I don't think anybody can say that they were 100% engaged 100% of the time… (C2)

Everyone having pen and paper to write things down, but having our own space to write things, a turn to speak and then write stuff down to be collected that might help (C1)

Box 8
Length of the session

I think the session was also too short for all the patients and carers to make a full contribution to a complex topic. (R4b)

…a little bit limited at the end because time was running out but again I don't think that was anyone's fault, it was just that that the topics were so interesting so that many people wished to contribute. I think in a way that's a sign of a very successful event. (C3)

…I tried to include everyone, but it was very difficult…(R5a)

…in order for everybody to be able to contribute to the discussion we had to really move forward quite quickly and so everybody could maybe say a few sentences and then we couldn't go really in depth. So, it was more like the start of a conversation… If we had slightly more time it would have to be useful for going a bit more in-depth. (R3a)

…to me it was really ending quite quickly, when we were really getting into it better as we got towards the end…I thought the discussion was getting much more animated as time went on. At the beginning it took a little bit of time to present what they were doing. (C5)

Because I feel that the time we had until we really got into the details of the projects, we ran out of time for that reason… maybe in the last three or four minutes, we reached basically the beginning of the discussion I wanted to have. Also, from my perspective in trying to put those objectives of my project in the most specific way and in lay terms, understandable way, I think by the time we bridge these initial difficulties we run out of time (R4a)

Yes, we were told to stop, so we could have gone for longer. Maybe part of it was that it took us a while to figure out what was going on… (C1)
