# Supplementary material for: Fleshing out the data: when epidemiological researchers engage with patients and carers. Learning lessons from a patient involvement activity
Source: BMJ Open. 2020 Sep 30;10(9):e036311. doi: 10.1136/bmjopen-2019-036311 (PMC7528373; doi:10.1136/bmjopen-2019-036311)
Supplement: Author's manuscript [file bmjopen-2019-036311.draft_revisions.pdf]

BMJ Open

BMJ Open is committed to open peer review. As part of this commitment we make the peer review history of every article we publish publicly available.

When an article is published we post the peer reviewers' comments and the authors' responses online. We also post the versions of the paper that were used during peer review. These are the versions that the peer review comments apply to.

The versions of the paper that follow are the versions that were submitted during the peer review process. They are not the versions of record or the final published versions. They should not be cited or distributed as the published version of this manuscript.

BMJ Open is an open access journal and the full, final, typeset and author-corrected version of record of the manuscript is available on our site with no access controls, subscription charges or pay-per-view fees (http://bmjopen.bmj.com).

If you have any questions on BMJ Open's open peer review process please email info.bmjopen@bmj.com

BMJ Open

Fleshing out the data - when epidemiological researchers engage with cancer patients and carers: learning lessons from a patient involvement activity

Journal:	*BMJ Open*
Manuscript ID	bmjopen-2019-036311.R1
Article Type:	Communication
Date Submitted by the Author:	24-Feb-2020

Complete List of Authors:	Morris, Melanie; London School of Hygiene and Tropical Medicine, NCDE<br>Alencar, Yuki; London School of Hygiene and Tropical Medicine, Department of Non-Communicable Disease Epidemiology<br>Rachet, Bernard; London School of Hygiene and Tropical Medicine,<br>Stephens, Richard; Co-Editor in Chief, Research Involvement and Engagement; Consumer Lead, NCRI<br>Coleman, Michel; London School of Hygiene and Tropical Medicine, Department of Non-communicable Disease Epidemiology

**Primary Subject Heading**:	Research methods
Secondary Subject Heading:	Communication, Epidemiology, Public health
Keywords:	STATISTICS & RESEARCH METHODS, EPIDEMIOLOGY, PUBLIC HEALTH

SCHOLARONE™
Manuscripts

I, the Submitting Author has the right to grant and does grant on behalf of all authors of the Work (as defined in the below author licence), an exclusive licence and/or a non-exclusive licence for contributions from authors who are: i) UK Crown employees; ii) where BMJ has agreed a CC-BY licence shall apply, and/or iii) in accordance with the terms applicable for US Federal Government officers or employees acting as part of their official duties; on a worldwide, perpetual, irrevocable, royalty-free basis to BMJ Publishing Group Ltd ("BMJ") its licensees and where the relevant Journal is co-owned by BMJ to the co-owners of the Journal, to publish the Work in BMJ Open and any other BMJ products and to exploit all rights, as set out in our licence.

The Submitting Author accepts and understands that any supply made under these terms is made by BMJ to the Submitting Author unless you are acting as an employee on behalf of your employer or a postgraduate student of an affiliated institution which is paying any applicable article publishing charge ("APC") for Open Access articles. Where the Submitting Author wishes to make the Work available on an Open Access basis (and intends to pay the relevant APC), the terms of reuse of such Open Access shall be governed by a Creative Commons licence – details of these licences and which Creative Commons licence will apply to this Work are set out in our licence referred to above.

Other than as permitted in any relevant BMJ Author's Self Archiving Policies, I confirm this Work has not been accepted for publication elsewhere, is not being considered for publication elsewhere and does not duplicate material already published. I confirm all authors consent to publication of this Work and authorise the granting of this licence.

For peer review only

Fleshing out the data - when epidemiological researchers engage with cancer patients and carers: learning lessons from a patient involvement activity

Melanie Morris,[1] Yuki Alencar,[1] Bernard Rachet,[1] Richard Stephens,[2] Michel P Coleman[1]

[1] Cancer Survival Group, Department of Non-communicable Disease Epidemiology, London School of Hygiene and Tropical Medicine, Keppel Street, London WC1E 7HT

[2] NCRI Consumer Forum, 2 Redman Place London E20 1JQ

Corresponding author:

yuki.alencar@lshtm.ac.uk
+44(0)20 7927 2551

Words: 3628 (excluding abstract, references, acknowledgments and supplementary materials)

Supplementary file:

Tables: 1

Figures: 2

Boxes: 8

Keywords: patient and public involvement, patient and public engagement, epidemiology, cancer, research, secondary data analysis, methods of engagement, evaluation

For peer review only

Abstract

Public and Patient Involvement and Engagement has become an essential element of health research, ensuring aims and outputs are worthwhile and relevant. However, research involving secondary data analyses does not present immediately obvious ways to involve patients and the public. Innovative approaches to ensure their involvement is meaningful and effective are required.

The Cancer Survival Group co-hosted a full-day meeting with the National Cancer Research Institute (NCRI) Consumer Forum – a group of cancer patients and carers. This included the Forum's "Dragons' Den": a small-group session in which their members provided insight, advice and ideas on current or planned research in the Cancer Survival Group.

We investigated this activity as an example of effective patient involvement, with the aim of developing broad recommendations to improve epidemiological/quantitative research by involving patients and carers as directly as possible.

In addition to quantitative data captured through evaluation forms filled in after the event, we used semi-structured interviews of a sample of participants to evaluate the effectiveness of the session and to learn lessons. The interviews were analysed to identify broad or recurrent themes and recommendations.

Feedback was overwhelmingly positive, and some impacts on the research projects were identified. Interviewees commented on overall expectations and experiences, as well as specifics of room layout, timing of the session, composition of groups, effectiveness of the facilitation and content of discussions.

We present a summary of our findings as a guide for other researchers, including recommendations for improvement gleaned from the interviews. The value to researchers of hosting and participating in such activities was clear. We were able to develop recommendations that should help to improve future events for ourselves and for others who wish to conduct similar activities, which in turn may lead to more concrete benefits for research and patients.

Article Summary – Strengths and limitations of this study

- **Innovative approaches for involving patients and the public are needed for epidemiological research to ensure it is meaningful and effective**
- **This novel format of involvement has been evaluated to provide insight and gather recommendations for improvements**
- **Comments on the room layout, session timing, composition of groups and effectiveness of facilitation led to lessons learned that will be implemented in the future**
- **More needs to be done to ensure a broader range of patients and the public participate in PPI activities, to ensure the experiences and views of all areas of society are present**
- **Findings can be used by other researchers in a variety of areas to establish effective forums for public and patient involvement (PPI)**

For peer review only

Introduction

The Cancer Survival Group (CSG) at the London School of Hygiene & Tropical Medicine has actively involved cancer patients and carers in its research for many years.[1] As members of the Group's Advisory Panels, they comment on research and propose ideas for further research or funding. In 2017 the CSG co-hosted an event with the NCRI Consumer Forum with whom we have a well-established relationship. This event included members of the Forum contributing to our research through discussion of our projects, in "Dragons' Den" style. This and other forms of patient involvement are common in other areas of research[2-5] but is not often attempted by epidemiological researchers.[6] Here, we set out the lessons learned, to help other researchers capitalise on the involvement of patients.

What is real patient involvement?

The involvement of patients, carers and the public[a] in health research helps to ensure that the research being planned and the approaches undertaken to deliver it are relevant to their needs and interests.[7-10] It also provides insights from the experience of people who use health services, or live with the condition in question. INVOLVE, the UK's national advisory group for public involvement in research, defines it as "research being carried out 'with' or 'by' members of the public rather than 'to', 'about' or 'for' them".[11 12] Patients and the public can offer new perspectives that researchers may be unable to provide, and can help improve the quality of the research.[7 10-12] They also highlight the wider consideration of making health research more transparent and publicly accountable, as well as empowering patients themselves.[13]

As epidemiologists, we may forget that each data point represents a person. We need to re-examine whether we are asking the right questions of the data – those that are most relevant to improving the health and wellbeing of patients. However, researchers who use routinely collected data, rather than from primary collection such as patient surveys, may find it challenging to see how they can involve the public and patients more effectively in their research.[14-16]

An example of cancer patient involvement

The Cancer Survival Group co-hosted a one-day symposium with the NCRI Consumer Forum, for over 50 external invitees including cancer patients and carers. Our aim was to showcase the Group's recent and current work to these guests, and to explain in detail how data are used in our work.

As part of the day, cancer patients and researchers participated in an interactive session: 'The Dragons' Den'. Closer in concept to break-out workshops than to focus groups, the idea of the NCRI Consumer Forum's Dragons' Den is to be a collaborative event in which patients are not just listening to presentations, but are equal participants in a round-table discussion; in which there is a shared desire to move research forward; and during which researchers can learn directly from patients' experiences and perspectives to shape and improve their research.[17]

Consumer Forum members had chosen one of five groups, each of which was led by a researcher in a discussion of a particular research question. These topics had been circulated in advance, with some of the questions the researchers wanted to explore (**Error! Reference source not found.**). Following a brief presentation of the research area/question, the researchers asked the members for their input,

---

[a] Hereafter, for brevity, we will use the term "patients" or "cancer patients" to refer to this group, which may in fact include patients, carers and members of the public.

For peer review only - http://bmjopen.bmj.com/site/about/guidelines.xhtml

advice and ideas around the planned research (Figure 1). The session was followed with plenary presentations and discussion, allowing us to capture the salient points of conversations that had taken place at each table, and to identify any broader or recurrent themes that emerged.

We describe the experience from this session to illustrate how researchers can interact effectively with patients and their representatives to improve research. We describe how the session was run, and the feedback and lessons we learned. We offer recommendations for how future sessions could be planned, and how this technique can be used to make the most of opportunities for collaboration.

Set-up

A large teaching room, with maximum capacity of 70 persons was set up with five tables seating up to 10 people each (Figure 2). On the day, 45 people attended, two thirds (64%) of whom had been NCRI Consumer Forum members for two years or more. Most had expressed a preference for a particular topic. Those who had not were randomly assigned to a table. Each table had a group of patients and two CSG researchers, one of whom acted as rapporteur. We scheduled 40 minutes for the session, followed by a 45-minute plenary session for feedback. These timings were based on the usual duration for Dragons' Den sessions.

Methods for evaluating the meeting

Forum members were initially sent feedback forms within a week of the meeting. The researchers were asked to provide notes and immediate reflections. We received both positive and negative comments (Appendix B). We have attempted to go beyond the usual "polite" feedback to understand what was good about the session, but also what was challenging, so that we could identify lessons that other researchers could implement in their interactions with patients.

In the second phase, semi-structured interviews with five patients and five researchers, one from each table, were carried out within six months of the meeting (Appendix C). These interviews were transcribed and thematically analysed, along with the notes collected immediately after the event.

This study was approved by the LSHTM Ethics Committee (ref. 14502)

Patient and Public Involvement

One of the co-authors (RS) is a patient representative and former Chair of the NCRI Consumer Forum of cancer patients and carers. The project idea was conceived together with RS and he was involved in the study concept and design.

Summary of reflections and feedback

In the following illustrative quotes, Consumer Forum members are coded C1-5 and researchers are coded R1-5, with 'a' and 'b' denoting the two researchers on each table. Patients with the same numbers as researchers were on the same table. More examples of participant responses for each section are in Appendix D.

Experience and Content

Overall experience (what did you get out of the session?)

Reflections on the overall experience were overwhelmingly positive. Both researchers and patients stated that the session had met or exceeded their expectations, that they learned something from being part of it, and that they would want to repeat it.

For peer review only

All the topics were relevant, it was well planned, relevant to patients with cancer, living with and beyond cancer… I would like to see it repeated because I think it was a very fruitful event. (C3)

However, there were also some comments on difficulties or limitations of the session.

Expectations / Importance of patient contribution

Both researchers and patients articulated the value of the contribution from members of the NCRI Consumer Forum. The patients and their carers felt that their input was valued, that they could improve the clarity of the research questions asked by the researchers, and help in considering the practicalities involved in the research.

Working together towards improving outcomes for patients is the best way to achieve reliable results… As a colleague on an equal basis everyone working side-by-side to discuss the research and receiving equal consideration it's so important I think to provide this opportunity. (C3)

Although it was clear that input from the patients had made a concrete difference to the researchers' work, some patients felt that more needed to be done to show them the value of their input after the event.

I don't know that I've heard very much what about what will actually happen, what the next steps are… (C2)

The researchers' responses focused on the new insights and perspectives that the patients had brought, including how it helps to remind us that there are real people represented by each data point.

Content of discussion

Inevitably, the content of the discussions varied greatly between groups. It was clear from the responses of both patients and researchers that some of the projects were less amenable to this format of discussion than others, for example when the project was primarily about methodological development.

There were a few steps that the Consumers needed to understand before they could really answer things that would be useful to me…all of these concepts were quite tricky to grasp. (R1a)

Where there was synergy, the discussion flowed easily, the time seemed too short and the suggestions made were fruitful and well received. At other tables, it took longer for mutual understanding to develop, although it was generally reached in the end.

Organisation of groups

The final allocation saw 6-8 patients at each table. Ultimately, 10-12 individuals participated in each group; including researchers, patients and, in some cases, other observers.

Composition of groups

Researchers and patients were generally positive about the size and overall composition of the groups. The size allowed for a variety of backgrounds to be represented at each table, which was viewed as beneficial to the discussions.

It was clear, though, that there is a happy medium for the number of participants that provides both diversity of experience and sufficient time for all participants to contribute to fruitful discussion.

I think it was fine. I wouldn't put more. But less is maybe too - you may not achieve what you want. I think you need the diversity, but not too many people. (R2a)

Due to the size and diversity of the groups in terms of experiences, one researcher felt that time was spent at the start in order for everyone to '*get on the same page*' *(R4a)*. However, it was also recognised by both researchers and patients, that there was a lack of diversity and representation of

certain demographic groups, because members of the NCRI Consumer Forum inevitably represent a sub-set of the population. NCRI Consumers are recruited in open competition, mainly to serve on academic research committees or other research groups working at a strategic level, and a successful applicant is required to have some prior knowledge or experience of cancer research.[18]

Facilitation of discussion

The researcher presenting the topic at each table facilitated the discussions. Feedback from the patients and other researchers on how that role was performed was generally positive, but it was suggested that combining facilitation with explaining the research ideas and the main goals of the session was too much, and that an 'independent' facilitator might be helpful. There was also a recommendation that it might be an advantage to have more senior moderators, who would feel more comfortable being directive in keeping the discussions on topic.

> *I think if maybe it had been a more senior person there…who is almost monitoring, keeping an eye on what's going on, giving a helping hand if somebody looks a little bit adrift, they could intervene at that point and get things back on track for them. (C5)*

One point identified for aiding facilitation was a suggestion that more structure to the questions might have helped direct the discussions. Clarifying at the outset what the main point is, where contributions would help, and which issues were in fact less important, may have made the session more fruitful.

Given that the researcher at each table was both presenting the research and facilitating the discussions, having a separate note-taker was appreciated. Another suggestion was to encourage others to make notes, and that the inclusion of visual aids, such as whiteboards or flip charts, might help to clarify the main issues, and focus and direct the discussions.

Layout and timing

Layout of the room

The impact of the layout of the room featured for both patients and researchers, who felt that the room was too small and the tables too close together. There was some concern that those with hearing difficulties were disadvantaged by the layout, and the patients, predominantly, felt that the discussions would have been better had the layout had been different.

> *…there were loads of tables in the one room. It was actually quite difficult to hear so that was frustrating in some ways (C2)*

Some also made recommendations about having each group in a separate room, although others felt the 'buzz' of the room helped create a good atmosphere.

Consideration of special needs

In their feedback, the patients gave some useful comments regarding consideration of the special needs of their group. These centred around two main aspects: firstly, the room layout impacting those with hearing impairments (as above). There was the practical suggestion that materials should have been provided for note-taking.

> *Everyone having pen and paper to write things down, but having our own space to write things, a turn to speak and then write stuff down to be collected that might help (C1)*

Secondly, to reduce the intensity and length of the day, especially considering the long-term health conditions of some of the patients, some of whom were still undergoing treatment.

For peer review only

Length of the session

The session was scheduled after the lunch break for 40 minutes, followed by a 45-minute plenary session. Given the length of time it took for each table to settle and begin the discussions, and after a few minutes of introduction and/or background to the topic by the researcher, there were roughly 30 minutes for open dialogue.

Many comments indicated that the time was too short, based partly on the numbers of patients at each table, but mainly on the complexity of the topics being presented.

> *…in trying to put those objectives of my project in the most specific way and in lay terms, understandable way, I think by the time we bridge these initial difficulties we run out of time (R4a)*

In a few groups it seemed that the discussions only made progress towards the end of the time allocated. Both patients and researchers commented that a substantial part of the time was taken at the start of the session to develop a good understanding of the project and where the researchers needed insight from the patients.

Summary of post-event evaluation by patients

Two-thirds (64%, 29/45) of patients responded to the request for evaluation immediately following the event. The Dragons' Den session was rated as the most informative session of the day by patients. In fact, 86% rated the group session informative for their "personal development as a consumer in cancer research" at some level, and almost three-quarters of attendees (72%) found the session useful for their role as an NCRI Consumer to some degree (Appendix B).

Overall, hosting of each table was considered effective (26/29, 90%), but half of respondents found it only slightly effective, with a few patients rating it neutral or ineffective. Most respondents found the event well organised (93%) and the venue location good (90%), but fewer found the room comfortable (59%).

Recommendations for the future

The lessons learned from the experience have helped inform the Cancer Survival Group's interactions with patients. We hope they will offer guidance to other researchers who want to arrange similar sessions. Although the epidemiological and methodological research in the Cancer Survival Group differs from that on which patients are more used to being consulted (e.g. designing clinical trials), the experience can be fruitful for both patients and researchers.

Suggestions for running a similar patient involvement session

Before the event:

Send detailed "pre-work" which has been piloted with one or two patients first, ideally co-written by them

Explain each study succinctly. Outline the specific questions that the researchers wish to explore. Encourage patients to send questions in advance to show the researchers what they might want to have clarified.

On the day:

Ensure enough space between tables so that all can be heard

For peer review only - http://bmjopen.bmj.com/site/about/guidelines.xhtml

Ensure the layout is appropriate for the size of groups. Seating arrangements will be dependent on the available space, but ideally the tables should be sufficiently well spaced that discussions at neighbouring tables are not a distraction.

While some groups may find background silence daunting and the buzz of conversation may help stimulate people to speak or make them feel more relaxed, for this group of experienced patients, most of whom were ready and willing to talk, a layout with more space would have been preferable. In another study which used a similar format of patient engagement, Hill et al[3] found that they successfully dealt with initial issues of noise by spacing the tables out in future events.

We would suggest only two to three groups (tables) per room if possible, so they can be spread as widely as possible. This allows a sense of privacy and ensures that everyone can hear and be heard.

We would recommend excluding others not directly involved in the session from the room (e.g. organisers, observers) as we found that these people add to the level of noise and can discourage openness.

Provide note-taking facilities and encourage their use

It is useful to provide pen and paper and/or post-it notes, and encourage notes and thoughts to be written down that can be shared with the researchers during and after the session.

A white-board or flip-chart for each group would enable diagrams and technical terms to be written up, and so that suggestions can be recorded for all to see. It is a good idea to photograph the boards or flip-charts at the end of the session, before they are taken down.

Allow plenty of time

If possible, take a whole day. Rather than having a one-off session, the day could be split into three discrete sessions, with breaks in between. Time can then be spent first on introductions, as well as allowing patients time to share their experiences. These all add to researchers' understanding, although they may not be directly relevant to the questions that ultimately need to be answered about each project.

Secondly, allow a generous amount of time for the introduction and initial discussion of the topic. This introductory session can focus on general questions the patients might have to clarify the work, or arising from their 'pre-work'. It should end with the researcher laying out the specific questions on which they would like to focus in the final discussion.

Lastly, run the Dragons' Den session, which should then focus on eliciting specific advice for the researchers to solve the problems they may have been having with study design, or on how patients' experiences could help improve their research question.

Have an independent facilitator for each group

To keep the session itself focused on the question in hand, we recommend having an 'independent' facilitator who is not directly involved in the research. This would preferably be a senior researcher with experience of chairing meetings who can be diplomatic but firm.

The benefits of an 'independent' facilitator would be in keeping the discussions focused, ensuring everyone is able and encouraged to speak, and summarising the main points to check for clarity.[3]

For peer review only

After the event:

Follow up with patients

As well as the usual evaluation forms, we recommend that researchers send follow-up notes to participants so that they can add further comments after having had a chance to reflect. This will add to the richness of what is gained from the day and use the patients' knowledge to the fullest extent.

It is also important to inform the people who have contributed to the research about what has happened as a result of their input and to let them know how the research has changed or developed as a result. This may be in the form of asking them to comment on research proposals based on the project discussed or in a brief report on its progress.

Remaining challenges:

Several challenges remain. The composition of patient groups was not fully representative of the cancer patient population. This was an observation also made in a systematic review of patient and public involvement in cancer research,[10] which found that PPI participants tend "to be well-educated female participants from ethnic majority groups" (p.14). Harder-to-reach groups should be invited, if possible, including more disadvantaged patients or their carers, those of different ethnicities and those with language barriers, or their representatives. By definition, it is difficult to include these groups, but efforts to approach them should be considered.

We would also like to be able to include patients with more special needs. Consideration should be given to the support such patients would need, and how it can best be provided.

The Cancer Survival Group is fortunate to have access to numerous teaching rooms, but space was still an issue. In some locations, space may be even more limited. We would still suggest attempting a Dragons' Den event, but perhaps for one or two groups at a time.

Conclusion

We have shared the lessons from one research group's experience of engaging with patients and their carers, in the hope of encouraging other researchers to carry out this kind of consultation, while starting from a more informed position.

The Dragons' Den process is extremely valuable to researchers engaged in all kinds of research, including epidemiological research based on routinely collected data. As well as receiving constructive advice on current research projects, it was also a chance for us to reconnect with the reason we do this research.

From the patients' perspective, participants were keen to contribute to work that would inform research and change policy. They emphasise the need for action and for everyone to "raise their voices" to work for easier access to data for epidemiological research, and to ensure that patients remain at the heart of all the research we do.

It was an invigorating and inspiring day, and we look forward to more engagement and involvement activities in the future.

Figure legends/captions

Figure 1: In the "Dragons' Den". Credit: Photograph by Simon Callaghan Photography

For peer review only

Figure 2: The Dragons' Den session under way, showing the layout of the room used. Credit: Photograph by Simon Callaghan Photography

For peer review only

References

1. Cancer Survival Group. About us: we work with people who have had cancer 2019 [updated 04/07/2019. Available from: https://csg.lshtm.ac.uk/what-we-do/#cancer accessed 06/08/2019 2019.

2. Hacker KE, Smith AB. Engaging Stakeholders and Patient Partners. *Surg Oncol Clin N Am* 2018;27(4):665-73. doi: 10.1016/j.soc.2018.05.007 [published Online First: 2018/09/15]

3. Hill K, Portman M, Tabi Z. Meet the researchers: an alternative method of engaging patients with research in mesothelioma. *Res Involv Engagem* 2018;4(1):33. doi: 10.1186/s40900-018-0119-x

4. Piil K, Jarden M. Patient involvement in research priorities (PIRE): a study protocol. *BMJ Open* 2016;6(5):e010615. doi: 10.1136/bmjopen-2015-010615 [published Online First: 2016/05/26]

5. Lee DJ, Avulova S, Conwill R, et al. Patient engagement in the design and execution of urologic oncology research. *Urol Oncol* 2017;35(9):552-58. doi: 10.1016/j.urolonc.2017.07.002 [published Online First: 2017/08/02]

6. Pattison N, Lee M. Two tribes coming together: patient and public involvement in cancer research. *Eur J Cancer Care (Engl)* 2014;23(1):1-2. doi: 10.1111/ecc.12178 [published Online First: 2014/01/01]

7. Stewart D, Wilson R, Selby P, et al. Patient and public involvement. *Ann Oncol* 2011;22 Suppl 7:vii54-vii56. doi: 10.1093/annonc/mdr427 [published Online First: 2011/11/09]

8. Gordon L, Dickinson A, Offredy M, et al. A research note on the benefit of patient and public involvement in research: The experience of prostate cancer patients regarding information in radiotherapy. *Radiography (London, England : 1995)* 2017;23(2):167-70. doi: 10.1016/j.radi.2017.02.004 [published Online First: 2017/04/10]

9. Ardron D, Kendall M. Patient and public involvement in health research: what is it, and why is it so important? *Int J Palliat Nurs* 2010;16(4):160-2. doi: 10.12968/ijpn.2010.16.4.47778 [published Online First: 2010/06/19]

10. Pii KH, Schou LH, Piil K, et al. Current trends in patient and public involvement in cancer research: A systematic review. *Health expectations : an international journal of public participation in health care and health policy* 2019;22(1):3-20. doi: 10.1111/hex.12841 [published Online First: 2018/11/01]

11. INVOLVE. Briefing notes for researchers: involving the public in NHS, public health and social care research. Eastleigh: INVOLVE, 2012.

For peer review only

12. INVOLVE. Briefing notes for researchers: What is public involvement in research? [Available from: https://www.invo.org.uk/posttyperesource/what-is-public-involvement-in-research/ accessed 07/08/2019.

13. Thompson J, Bissell P, Cooper CL, et al. Exploring the impact of patient and public involvement in a cancer research setting. *Qual Health Res* 2014;24(1):46-54. doi: 10.1177/1049732313514482 [published Online First: 2013/11/28]

14. Howe A, Mathie E, Munday D, et al. Learning to work together - lessons from a reflective analysis of a research project on public involvement. *Res Involv Engagem* 2017;3:1. doi: 10.1186/s40900-016-0051-x [published Online First: 2017/10/25]

15. Ocloo J, Matthews R. From tokenism to empowerment: progressing patient and public involvement in healthcare improvement. *BMJ Qual Saf* 2016;25(8):626-32. doi: 10.1136/bmjqs-2015-004839 [published Online First: 2016/03/20]

16. Puts MTE, Sattar S, Ghodraty-Jabloo V, et al. Patient engagement in research with older adults with cancer. *J Geriatr Oncol* 2017;8(6):391-96. doi: 10.1016/j.jgo.2017.05.002 [published Online First: 2017/06/11]

17. National Cancer Research Institute (NCRI). A 'Dragon's Den' workshop to improve research through Consumer involvement 2018 [updated 31/01/2018. Available from: https://www.ncri.org.uk/case-study/a-dragons-den-workshop-to-improve-research-through-consumer-involvement/.

18. NCRI Consumer Forum. Role Profile for Consumer Members of NCRI Clinical Studies Groups. http://www.ncri.org.uk/wp-content/uploads/2017/02/CSG-Consumer-Role-Profile-PDF-2017.pdf, 2017.

For peer review only

Author Statement

All authors conceived and planned the study. **MM** conducted and transcribed the interviews, gathered and analysed the data. **MM** and **YA** summarised the data, reviewed the literature and wrote the manuscript. **RS, MPC** and **BR** provided comments on the manuscript and all authors have seen and approved the final version of the manuscript for publication.

Data sharing statement

No additional data available.

Acknowledgements

We gratefully acknowledge all the participants of the Dragons' Den session, in particular those who were interviewed after the event. We also acknowledge Dr Edmund Njeru Njagi for his contribution to the collation of feedback after the event and comments on the initial paper outline.

Conflicts of interests

The authors declare no conflicts of interests.

Funding

YA is supported by Cancer Research UK grant number C7923/A18525

For peer review only

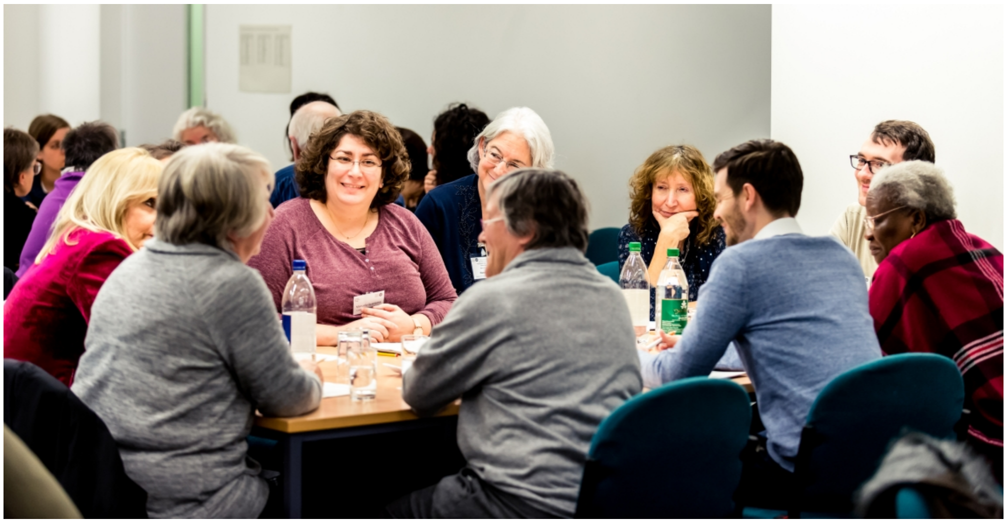

Figure 1: In the “Dragons’ Den”. Credit: Photograph by Simon Callaghan Photography

342x176mm (200 x 200 DPI)

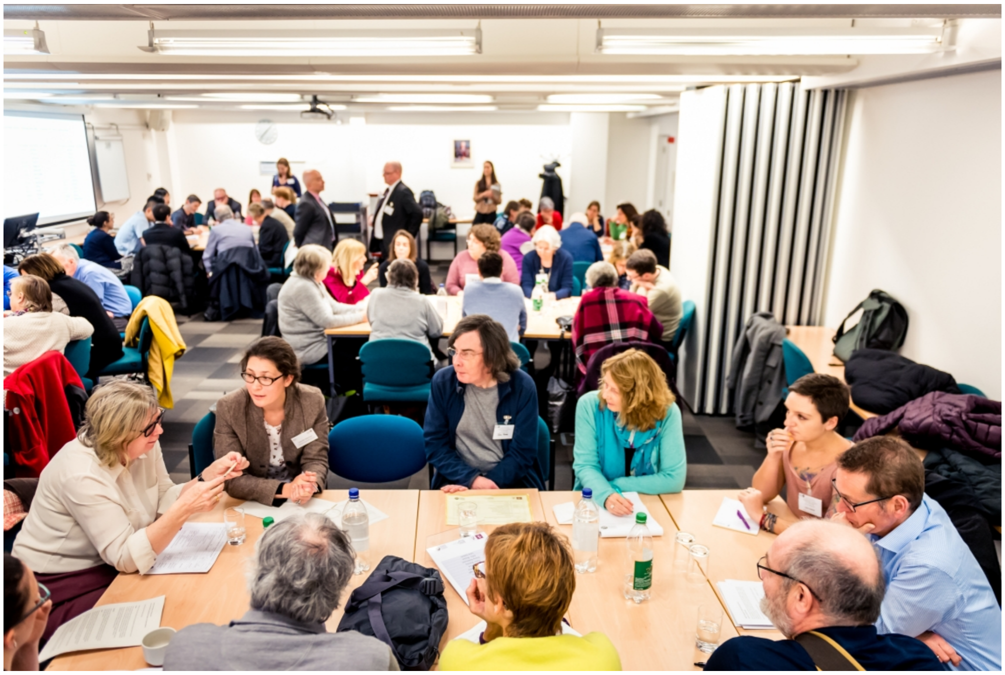

Figure 2: The Dragons' Den session under way, showing the layout of the room used. Credit: Photograph by Simon Callaghan Photography

342x228mm (200 x 200 DPI)

Appendix A: Topics of Dragon's Den tables (Feb 2017)

#	Table Topic	Research summary
**1**	Opportunities for reducing emergency cancer diagnosis: the role of comorbidities in influencing help-seeking for cancer symptoms and access to diagnostic investigations	We aim to examine the clinical events during the months before a cancer diagnosis in order to identify opportunities to diagnose cancer earlier and reduce emergency cancer diagnoses. In particular, we want to better understand why patients with a long-standing health problem (comorbidity) often have a higher risk of delayed cancer diagnosis and emergency presentation.
**2**	Examining inequalities in cancer survival for colon cancer patients living in London and treated with surgery in a London hospital	I am looking into identifying the main drivers of these differences in survival including factors such as: CCG of residence, hospital of surgery, socio-economic background, stage at diagnosis, specialised colorectal-surgeon, discussion of treatment plan in a MDT.
**3**	Evaluation of public health interventions	We aim to evaluate public health cancer policies soon after their implementation or even before their implementation. Using the technique of 'scenario modelling', by which we artificially change the composition of our cohorts of patients and how they are managed, to reflect the likely impact of a policy, we would like to look at the effect of those policies on cancer survival and other derived measures.
**4**	Factors influencing decision-making in cancer care.	These studies aim to explore which factors influence doctors' and patients' decision-making regarding the management of an aggressive type of Non-Hodgkin Lymphoma called Diffuse Large B-Cell Lymphoma (DLBCL) and/or bowel cancer. In particular, we are interested in people's attitude towards clinicians' management of elderly cancer patients.

For peer review only

**5**	Inequalities in cancer management and survival: NHS processes and/or lack of resources?	Some groups of cancer patients, for example from the more deprived categories, experienced lower survival from their cancer. It has been shown that these patients do not receive the most optimal management (including diagnostic investigation and treatment), and their higher proportion of comorbidity and more advanced stage at diagnosis would explain only part of these inequalities in cancer management. The reasons these patients are, for example, more likely to be managed in non-specialised hospitals or to be operated by less experienced surgeons are not understood. We aim to investigate the role of the organisation (processes) of the NHS and its disparities in resources, in these inequalities in cancer management and cancer survival.

For peer review only

Appendix B: Evaluation of relevant parts of the day given within a week of the event

	No new information	Reinforced existing information	Slightly informative	Informative	Extremely informative
Group session	0 (0.0%)	4 (13.8%)	8 (27.6%)	4 (13.8%)	13 (44.8%)
Plenary feedback session	0 (0.0%)	4 (13.8%)	6 (20.7%)	6 (20.7%)	13 (44.8%)
	**Topic not relevant to me**	**Relevant but not useful**	**Slightly useful**	**Useful**	**Extremely Useful**
Group session	1 (3.4%)	6 (20.7%)	5 (17.2%)	6 (20.7%)	10 (34.5%)
Plenary feedback session	1 (3.4%)	4 (13.8%)	7 (24.1%)	7 (24.1%)	9 (31.0%)
	**Ineffective**	**Neither effective nor ineffective**	**Slightly effective**	**Very effective**	**Exceptional**
Table hosting	2 (6.9%)	1 (3.4%)	13 (44.8%)	9 (31.0%)	4 (13.8%)
	**Unsatisfactory**	**Neither satisfactory or unsatisfactory**	**Slightly Satisfactory**	**Very satisfactory**	**Exceptional**
Event organisation	0 (0.0%)	1 (3.4%)	1 (3.4%)	21 (72.4%)	6 (20.7%)
Building and location	0 (0.0%)	0 (0.0%)	2 (6.9%)	20 (69.0%)	6 (20.7%)
Room and comfort	1 (3.4%)	2 (6.9%)	9 (31.0%)	12 (41.4%)	5 (17.2%)

For peer review only

Appendix C: Semi-structured interview questions

Interview questions for consumers

1. What were you hoping to gain from participating in the Dragon's Den session?

2. Did it meet your expectations?

3. Do you feel your comments were listened to and taken on board?
    a. How did the researcher make that possible?

    OR b. What was it about the session that meant that didn't happen?

4. Did you feel that the feedback provided by the consumers will make a positive difference to the project?

5. Did you feel that there was enough opportunity for questions and discussion?

6. Was the session the right length/layout/number of participants?

7. Do you have any comments or suggestions for improvements that we could make to the session in order to better meet the needs of researchers and/or consumers?

8. Any other comments?

Interview questions for researchers

1. What were you hoping to gain from presenting at the Dragon's Den session?

2. Were you able to achieve those objectives?

3. Did the session meet your expectations?

4. Do you think the feedback provided by the consumers will make a positive difference to the project?
    a. In what way?
    b. Or why not?

5. What do you think the consumers got out of the session?

6. Did you feel that there was enough opportunity for questions and discussion?

7. Was the session the right length/layout/number of participants?

8. Do you have any comments or suggestions for improvements that we could make to the session in order to better meet the needs of researchers and/or consumers?

9. Any other comments?

For peer review only

Appendix D

Box 1

Overall experience (what did you get out of the session?)

It's an opportunity to hear what other people think about something, expanding my own learning and it gives me more experience that I can draw on in the future…So for me it's about trying to get my bit across, listen to everyone else, and try to learn a bit more, it's about a learning process (C2)

All the topics were relevant, it was well planned, relevant to patients with cancer, living with and beyond cancer... There is a feeling of altruism wanting to give back and help someone's knowledge and experience so perhaps this information will help others being treated in the future…I would like to see it repeated because I think it was a very fruitful event. (C3)

I enjoyed it very much and got a lot out of it. (C4)

I was thrilled at that opportunity to discuss with Consumers...it was a great experience, and I wish we had more opportunities to share about the work we do in that context (R1a)

I thought it was a very useful exercise and patient representatives were really active and each of them really had something to say on the topic. So, I think it was a really useful interaction with patients. (R3a)

It was the first time I was involved in the Dragons' Den, I would like to definitely to repeat it (R4a)

I was excited to get some information from people with first-hand experience of cancer. (R4b)

On the actual day, the sessions were largely as I had expected them to be. The participants were very…keen to share their views and experiences, which were very insightful. (R5b)

It was a lot more difficult than I thought. I didn't really know what we were there for, so it was a bit challenging at times. (C1)

I was also aware that there may not be easy answers to our research question. The session was interesting but also quite difficult. (R4b)

Because it went so well actually I wanted to go beyond to have more information, to discuss more etc, and that was quite frustrating. And for everyone I think. (R2a)

I was a bit stressed, to start with, because I had no clue…if I would have to do the talking non-stop or the listening non-stop, and I think it went well overall, but it kind of got out of control…so in the end, I was happy when it finished, it was enough. (R1a)

Box 2

Expectations / Importance of patient contribution

Working together towards improving outcomes for patients is the best way to achieve reliable results… the genuine information can come from those who have experienced the situation undergoing that, dealing with things…As a colleague on an equal basis everyone working side-by-side to discuss the research and receiving equal consideration it's so important I think to provide this opportunity. (C3)

I believe the answers provided by the Consumers will provide more clarity on the question and the practicalities to be considered in the research. (C4)

You don't always know whether you are really being listened to or not because people are polite generally speaking, so therefore they sometimes can maybe appear to be interested. You don't know when they go away, whether they actually do anything with it or not. But I was aware in this case that they did, so that was very positive…I mean the researchers have a very good idea of the overview of what they want to get to, but sometimes it's the practical issues that the PPI [Public and Patient Involvement] can throw in. (C5)

It obviously did turn out to be pertinent. They showed that by their response at the time and I thought that those issues were incorporated in the grant proposal that we got to read later on...So, it was actually made very obvious that they did take that on board… (C5)

For peer review only

I don't know that I've heard very much what about what will actually happen, what the next steps are…it might be useful if there's something you could pass around at the end of the session to say "This is where this particular project is, here's the written summary of what you said" just some way of feeding that back. It was all said verbally. (C2)

So, I was hoping to get somehow, an insider's view of what matters to the patients really in terms of, or what they think could improve survival, somehow, stuff that could be addressed by policy makers. (R1a)

I think that it's useful to have the views from the patients to know that you're on the right track…And I hope they see that we value their point of view and their opinion and their contribution and how we have the need to hear what they think about the issues we are trying to study. (R3a)

The patients were well informed; their answers were precise, informative, and not just anecdotal. (R2b)

It brings a different perspective and different insights. (R4a)

It was touching to listening to their experiences and I appreciated the fact that most people were open about their personal experiences. (R1b)

Like all these things, they seem very obvious to them, but of course you won't get that sense from looking at registry data... It was a good reminder for us that we sit in front of the computer and it loses that personal touch…it felt really intense to hear their experience. (R5a)

Box 3

Content of discussion

There were a lot of us that for a while didn't really understand what was being asked of us and how we could help with the research. We did eventually but there was quite a while where we weren't quite sure for a while what exactly we were supposed to be inputting…it took us a while to figure out what the point to it was exactly. That wasn't clear at all, it was the nature of the study I guess (C1)

I think at the beginning of the session, it wasn't as clear as it could be exactly what the researcher was wanting to ask us. (C4)

There were sort of two bits to it, so it would have been easier if there had only been one piece rather than two angles. (C5)

There were a few steps that the Consumers needed to understand before they could really answer things that would be useful to me…So all of these concepts were quite tricky to grasp, and I discovered it as we were talking with the patients as well…It's more of a stats [project] so for me it was hard to translate into words and applications, and for them it was hard to get the stats concepts behind…I would have picked another subject I guess. (R1a)

I presented a very broad project…but I think it was too broad. (R4a)

But after a bit of fumbling we were definitely able to come up with a lot of stuff (C1)

We didn't have enough time to discuss: "So what? What can we do etc?" What I wanted to discuss, "Now there are some initiatives to try tackle that, or maybe not, we don't know - what do you think? Do you think it worsens the situation or does it help?" And we didn't have time and I think it would have been very useful. (R2a)

Box 4

Composition of groups

The number of participants, I think it was a good number and it was useful to have to people from different experiences, ages, and carers and patients and so I think it was a good mix and the number was right... And the fact that they were men and women, different age groups, I think that was very interesting, carers and patient[s]…If there was a bit more variety in terms of background of the participants, it would maybe make the discussion even richer, even open up new insights…The only downside I think was that these were all people who were highly engaged, eloquent, had very clear views, had thought about it a lot. So, when we

very briefly talked about the issue of lower SES, obviously there wasn't anybody there representing those groups so that was a bit of a limitation. (R3a)

I thought the number of participants was good, I thought the breadth of experience of the participants was good… In terms of representation, that was very good (C4)

I think it was fine. I wouldn't put more. But less is maybe too - you may not achieve what you want. I think you need the diversity, but not too many people. (R2a)

I think less people, not many less people but a few less people around the table would have been good, especially for the length of time… (C1)

…we are not representative of the population. It's the same with most patient reps - it tends to be…well educated white middle class with sharp elbows…they have resources in terms of time and education, can put some time aside and can make a difference, but we're not necessarily reflecting the population that we should reflect, given that cancer is a disease, certain cancers are more prevalent within certain communities. (C2)

I went away with the feeling that this is such a selected group … they are active patients because they're involved in these sort of things... Because they are there, and they are patients who they know that they can be useful for research (R5a)

Box 5

Facilitation of discussion

I thought [the researcher] was an excellent facilitator. Fortunately, Consumers are very talkative, so it's quite a job to not just comment and keep things going but try and keep things within the thing that you're supposed to be talking, trying to keep things moving along and I think that was done well (C2)

Yes – it was an open discussion, and everyone at the table could contribute and those that weren't able to easily contribute for example, having hearing difficulties, everyone was made to feel that had the same input as others, in a position where they were able to contribute appropriately…, I do remember the researcher being engaged with everybody. (C4)

[The lead researcher] did a good job in describing the topic and engaging members and handled the questions well by triggering different conversations (R1b)

I think it was tricky as the presenter to be presenting my topic and also moderating the discussion because I don't have those skills, of a moderator, and I also had to get my point across and I had to welcome everyone's suggestions so that everyone would feel free to discuss and that was quite tricky…It was really good that I didn't have to take the notes, because I think that would have been really hard… I made sure I was engaged in making eye contact. (R1a)

I think if maybe it had been a more senior person there … who is almost monitoring keeping an eye on what's going on, giving a helping hand if somebody looks a little bit adrift, they could intervene at that point and get things back on track for them… if you can use a whiteboard or a flipchart, not to make it too long, but a picture always can speak a thousand words and if you can draw a little diagram or a couple of words that sound very difficult, write them up so that people are clear what you're talking about. It makes it a little bit easier both for them to explain and for the other people to understand (C5)

[A consumer] was the one that actually tried to somehow focus the others, saying, "No, wait a minute, we're here to discuss this and this," because obviously [we were] not reaching that common point of understanding for all…I think the key is the structuring a priori because that would avoid … cross talking because it's natural, it's open and that's what happens (R4a).

Box 6

Layout of the room

The acoustics weren't very good, and the layout of the table was such that sometimes it was hard to hear what was being said by maybe another discussant at the far end...Either the room needed to be a little bit

bigger, so that the tables were more spaced out, which is what we would generally have. Or needed to be in a separate little sub room where you could talk a little bit more readily. The main thing I felt really was that the room that was used wasn't terribly great, it was squashed and too noisy and that caused issues (C5)

…maybe separate rooms with complete silence around would have helped a little bit. (R1a)

…there were loads of tables in the one room. It was actually quite difficult to hear so that was frustrating in some ways because on that small table if there had been more space a lot of us would have got a lot more out of that particular meeting (C2)

…it's always that thing of hearing what's being said at other tables, adjacent tables, but that's really environmental and beyond the control of the school itself. I do wonder if other participants…might feel a little hesitant about expressing, describing their personal situation…(C3)

For me I'm a little deaf, then it was hard because we had a lot of tables, it was a little noisy…we would need probably more space because the acoustics are not really good…You know, in a way it's nice to see this, I think it's - you know you are part of a big event in a way. It can be quite nice. While if you are all in different rooms… (R2a)

The room was loud, and a participant complained they could not hear or contribute to our discussion. (R4b)

Box 7

Consideration of special needs

There were a couple of people at our table who were a little older. And because [of that] they weren't as quick to pick up in terms of hearing, and they found the noisy background a bit of a distraction at times. (C5)

I think the noise could be a problem for anyone with hearing difficulties…there was always a low buzz of conversation which is always natural when people are speaking (C3)

…because there were a couple of people at the tables who had hearing issues, and they did find it hard to hear, so that could have been done better. (C4)

It was quite a full-on day, all day long and I don't think anybody can say that they were 100% engaged 100% of the time… (C2)

Everyone having pen and paper to write things down, but having our own space to write things, a turn to speak and then write stuff down to be collected that might help (C1)

Box 8

Length of the session

I think the session was also too short for all the patients and carers to make a full contribution to a complex topic. (R4b)

…a little bit limited at the end because time was running out but again I don't think that was anyone's fault, it was just that that the topics were so interesting so that many people wished to contribute. I think in a way that's a sign of a very successful event. (C3)

…I tried to include everyone, but it was very difficult…(R5a)

…in order for everybody to be able to contribute to the discussion we had to really move forward quite quickly and so everybody could maybe say a few sentences and then we couldn't go really in depth. So, it was more like the start of a conversation… If we had slightly more time it would have to be useful for going a bit more in-depth. (R3a)

…to me it was really ending quite quickly, when we were really getting into it better as we got towards the end…I thought the discussion was getting much more animated as time went on. At the beginning it took a little bit of time to present what they were doing. (C5)

For peer review only

Because I feel that the time we had until we really got into the details of the projects, we ran out of time for that reason… maybe in the last three or four minutes, we reached basically the beginning of the discussion I wanted to have. Also, from my perspective in trying to put those objectives of my project in the most specific way and in lay terms, understandable way, I think by the time we bridge these initial difficulties we run out of time (R4a)

Yes, we were told to stop, so we could have gone for longer. Maybe part of it was that it took us a while to figure out what was going on… (C1)

For peer review only

BMJ Open

Fleshing out the data - when epidemiological researchers engage with cancer patients and carers: learning lessons from a patient involvement activity

Journal:	*BMJ Open*
Manuscript ID	bmjopen-2019-036311.R2
Article Type:	Communication
Date Submitted by the Author:	11-Aug-2020
Complete List of Authors:	Morris, Melanie; London School of Hygiene & Tropical Medicine, Cancer Surivival Group, NCDE<br>Alencar, Yuki; London School of Hygiene and Tropical Medicine, Department of Non-Communicable Disease Epidemiology<br>Rachet, Bernard; London School of Hygiene and Tropical Medicine,<br>Stephens, Richard; Research Involvement and Engagement, Co-Editor in Chief, Research Involvement and Engagement ; National Cancer Research Institute, Consumer Lead<br>Coleman, Michel; London School of Hygiene & Tropical Medicine, Cancer Survival Group, Department of Non-communicable Disease Epidemiology
**Primary Subject Heading**:	Research methods
Secondary Subject Heading:	Communication, Epidemiology, Public health
Keywords:	STATISTICS & RESEARCH METHODS, EPIDEMIOLOGY, PUBLIC HEALTH

SCHOLARONE™
Manuscripts

I, the Submitting Author has the right to grant and does grant on behalf of all authors of the Work (as defined in the below author licence), an exclusive licence and/or a non-exclusive licence for contributions from authors who are: i) UK Crown employees; ii) where BMJ has agreed a CC-BY licence shall apply, and/or iii) in accordance with the terms applicable for US Federal Government officers or employees acting as part of their official duties; on a worldwide, perpetual, irrevocable, royalty-free basis to BMJ Publishing Group Ltd ("BMJ") its licensees and where the relevant Journal is co-owned by BMJ to the co-owners of the Journal, to publish the Work in BMJ Open and any other BMJ products and to exploit all rights, as set out in our licence.

The Submitting Author accepts and understands that any supply made under these terms is made by BMJ to the Submitting Author unless you are acting as an employee on behalf of your employer or a postgraduate student of an affiliated institution which is paying any applicable article publishing charge ("APC") for Open Access articles. Where the Submitting Author wishes to make the Work available on an Open Access basis (and intends to pay the relevant APC), the terms of reuse of such Open Access shall be governed by a Creative Commons licence – details of these licences and which Creative Commons licence will apply to this Work are set out in our licence referred to above.

Other than as permitted in any relevant BMJ Author's Self Archiving Policies, I confirm this Work has not been accepted for publication elsewhere, is not being considered for publication elsewhere and does not duplicate material already published. I confirm all authors consent to publication of this Work and authorise the granting of this licence.

For peer review only

Fleshing out the data - when epidemiological researchers engage with cancer patients and carers: learning lessons from a patient involvement activity

Melanie Morris,[1] Yuki Alencar,[2] Bernard Rachet,[2] Richard Stephens,[3] Michel P Coleman[1]

[1] Cancer Survival Group, Department of Non-communicable Disease Epidemiology, London School of Hygiene and Tropical Medicine, Keppel Street, London WC1E 7HT

[2] Department of Non-communicable Disease Epidemiology, London School of Hygiene and Tropical Medicine, Keppel Street, London WC1E 7HT

[3] NCRI Consumer Forum, 2 Redman Place London E20 1JQ

Corresponding author:

Yuki Alencar

yuki.alencar@lshtm.ac.uk
+44(0)20 7927 2551

Words: 3628 (excluding abstract, references, acknowledgments and supplementary materials)

Supplementary file:

Tables: 0

Figures: 2

Boxes: 8

Keywords: patient and public involvement, patient and public engagement, epidemiology, cancer, research, secondary data analysis, methods of engagement, evaluation

For peer review only

Abstract

Public and Patient Involvement and Engagement (PPI) has become an essential element of health research, ensuring aims and outputs are worthwhile and relevant. However, research involving secondary data analyses does not present immediately obvious ways to involve patients and the public. Innovative approaches to ensure their involvement is meaningful and effective are required.

The Cancer Survival Group co-hosted a full-day meeting with the National Cancer Research Institute (NCRI) Consumer Forum – a group of cancer patients and carers. This included the Forum's "Dragons' Den": a small-group session in which their members provided insight, advice and ideas on current or planned research in the Cancer Survival Group.

We investigated this activity as an example of effective patient involvement, with the aim of developing broad recommendations to improve epidemiological/quantitative research by involving patients and carers as directly as possible.

In addition to quantitative data captured through evaluation forms completed after the event, we used semi-structured interviews of a sample of participants to evaluate the effectiveness of the session and to learn lessons. The interviews were analysed to identify broad or recurrent themes and recommendations.

Feedback was overwhelmingly positive, and some impacts on the research projects were identified. Interviewees commented on overall expectations and experiences, as well as specifics of room layout, timing of the session, composition of groups, effectiveness of the facilitation and content of discussions.

We present a summary of our findings as a guide for other researchers, including recommendations for improvement gleaned from the interviews. The value to researchers of hosting and participating in such activities was clear. We developed recommendations that should help to improve future events for ourselves and for others who wish to conduct similar activities, which in turn may lead to more concrete benefits for research and patients.

Article Summary – Strengths and limitations of this study

- **Innovative approaches for involving patients and the public are needed for epidemiological research to ensure it is meaningful and effective**
- **This novel format of involvement has been evaluated to provide insight and gather recommendations for improvements**
- **Comments on the room layout, session timing, composition of groups and effectiveness of facilitation led to lessons learned that will be implemented in the future**
- **Findings can be used by other researchers in a variety of areas to establish effective forums for public and patient involvement (PPI)**
- **More needs to be done to ensure a broader range of patients and the public participate in PPI activities, to ensure the experiences and views of all sectors of society are included**

For peer review only

Introduction

The Cancer Survival Group (CSG) at the London School of Hygiene & Tropical Medicine has actively involved cancer patients and carers in its research for many years.[1] As members of the Group's Advisory Panels, they comment on research and propose ideas for further research or funding. In 2017 the CSG co-hosted an event with the NCRI Consumer Forum with whom we have a well-established relationship. This event included members of the Forum contributing to our research through discussion of our projects, in "Dragons' Den" style (from the well-known television programme), in which the patients or public are the "dragons" to whom the researchers "pitch" their research ideas. This and other forms of patient involvement are common in other areas of research,[2-5] but is not often attempted by epidemiological researchers.[6] Here, we set out the lessons learned, to help other researchers capitalise on the involvement of patients.

What is real patient involvement?

The involvement of patients, carers and the public[a] in health research helps to ensure that the research being planned and the approaches undertaken to deliver it are relevant to their needs and interests.[7-10] It also provides insights from the experience of people who use health services, or live with the condition in question. INVOLVE, the UK's national advisory group for public involvement in research, defines it as "research being carried out 'with' or 'by' members of the public rather than 'to', 'about' or 'for' them".[11 12] Patients and the public can offer new perspectives that researchers may be unable to provide, and can help improve the quality of the research.[7 10-12] They also highlight the importance of making health research more transparent and publicly accountable, as well as empowering patients themselves.[13]

As epidemiologists, we try not to forget that each data point represents a person, but we need to re-examine whether we are asking the right questions of the data – those that are most relevant to improving the health and wellbeing of patients. Researchers who carry out secondary analysis of routinely collected data, rather than from primary data collection such as patient surveys, may find it challenging to involve patients more effectively in their research.[14-16]

In this communication piece, we outline what we learned from running a patient involvement activity. This was not intended to be a research project in itself, but we offer here some reflections on the experience that we hope will inform and benefit other researchers.

An example of cancer patient involvement

The Cancer Survival Group co-hosted a one-day symposium with the NCRI Consumer Forum, for over 50 external invitees including cancer patients and carers. Our aim was to showcase the Group's recent and current work to these guests, and to explain in detail how data are used in our work.

As part of the day, cancer patients and researchers participated in an interactive session: The Dragons' Den. Closer in concept to break-out workshops than to focus groups, the idea of the NCRI Consumer Forum's Dragons' Den is to be a collaborative event in which patients are not just listening to presentations, but are equal participants in a round-table discussion; in which there is a shared desire

[a] Hereafter, for brevity, we will use the term "patients" or "cancer patients" to refer to this group, which may include patients, carers and members of the public.

For peer review only

to move research forward; and during which researchers can learn directly from patients' experiences and perspectives to shape and improve their research.[17]

Consumer Forum members had chosen one of five groups, each of which was led by a researcher in a discussion of a particular research question. These topics had been circulated in advance, with some of the questions the researchers wanted to explore (**Error! Reference source not found.**). Following a brief presentation of the research area/question, the researchers asked the members for their input, advice and ideas around the planned research (Figure 1). The session was followed by a short plenary session in which a patient representative from each table presented the main points discussed, allowing us to capture the salient points of conversations that had taken place at each table, and to identify any broader or recurrent themes that emerged on the day.

We describe the experience from this session to illustrate how researchers can interact effectively with patients and their representatives to improve research. We describe how the session was run, and the feedback and lessons we learned. We offer recommendations for how future sessions could be planned, and how this technique can be used to make the most of opportunities for collaboration.

Set-up

A large teaching room with maximum capacity of 70 persons was set up with five tables seating up to 10 people each (Figure 2). On the day, 45 people attended, two thirds (64%) of whom had been NCRI Consumer Forum members for two years or more. Most had expressed a preference for a particular topic. Those who had not were randomly assigned to a table. Each table had a group of patients and two CSG researchers, one of whom acted as rapporteur. We scheduled 40 minutes for the session, followed by a 45-minute plenary session for feedback. These timings were based on the usual duration for Dragons' Den sessions.

Methods for evaluating the meeting

Forum members were initially sent feedback forms within a week of the meeting. The researchers were asked to provide notes and immediate reflections. We received both positive and negative comments (Appendix B). We have attempted to go beyond the usual "polite" feedback to understand what was good about the session, but also what was challenging, so that we could identify lessons that other researchers could implement in their interactions with patients.

In the second phase, semi-structured interviews with five patients and five researchers, one from each table, were carried out within six months of the meeting (Appendix C). These interviews were transcribed verbatim and thematically analysed, along with the notes collected immediately after the event.

The thematic analysis of the transcripts followed the stages of data familiarisation, coding, and theme refinement. Transcripts were read, cleaned and re-read by two authors [MM, YA]. The texts were coded by individual researchers, who assigned a tentative theme to each meaningful phrase. Themes were then discussed, grouped together and re-coded into overarching themes which are summarised below.[18 19]

This study was approved by the LSHTM Ethics Committee (ref. 14502)

Patient and Public Involvement

One of the co-authors (RS) is a patient representative and former Chair of the NCRI Consumer Forum of cancer patients and carers. The project idea was conceived together with RS and he was involved in the study concept and design.

For peer review only - http://bmjopen.bmj.com/site/about/guidelines.xhtml

Summary of reflections and feedback

The following seven themes emerged from analysis of the interview data: overall experience, expectations and importance of patient contribution; content of the discussion; composition of groups; facilitation of discussion; layout, including consideration of special needs, and timing.

In the following illustrative quotes, Consumer Forum members are coded C1-5 and researchers are coded R1-5, with ‘a’ and ‘b’ denoting the two researchers on each table. Patients with the same numbers as researchers were on the same table. More examples of participant responses for each section are in Appendix D.

Experience and Content

Overall experience (what did you get out of the session?)

Reflections on the overall experience were overwhelmingly positive. Both researchers and patients stated that the session had met or exceeded their expectations, that they learned something from being part of it, and that they would want to repeat it.

> *All the topics were relevant, it was well planned, relevant to patients with cancer, living with and beyond cancer… I would like to see it repeated because I think it was a very fruitful event. (C3)*

However, there were also some comments on difficulties or limitations of the session (see supplementary file Appendix D, Box 1).

Expectations / Importance of patient contribution

Both researchers and patients articulated the value of the contribution from members of the NCRI Consumer Forum. The patients and their carers felt that their input was valued, that they could improve the clarity of the research questions asked by the researchers, and help in considering the practicalities involved in the research.

> *Working together towards improving outcomes for patients is the best way to achieve reliable results… As a colleague on an equal basis everyone working side-by-side to discuss the research and receiving equal consideration it's so important I think to provide this opportunity. (C3)*

Although it was clear that input from the patients had made a concrete difference to the researchers’ work, some patients felt that more needed to be done to show them the value of their input after the event.

> *I don't know that I've heard very much what about what will actually happen, what the next steps are… (C2)*

The researchers’ responses focused on the new insights and perspectives that the patients had brought, including how it helps to remind us that there are real people represented by each data point (see supplementary file Appendix D, Box 2).

Content of discussion

Inevitably, the content of the discussions varied greatly between groups. It was clear from the responses of both patients and researchers that some of the projects were less amenable to this format of discussion than others, for example when the project was primarily about methodological development.

> *There were a few steps that the Consumers needed to understand before they could really answer things that would be useful to me…all of these concepts were quite tricky to grasp. (R1a)*

For peer review only

Where there was synergy, the discussion flowed easily, the time seemed too short, and the suggestions made were fruitful and well received. At other tables, it took longer for mutual understanding to develop, although it was generally reached in the end (supplementary file Appendix D, Box 3).

Organisation of groups

The final allocation saw 6-8 patients at each table. Ultimately, 10-12 individuals participated in each group, including researchers, patients and, in some cases, other observers.

Composition of groups

Researchers and patients were generally positive about the size and overall composition of the groups. The size of each group allowed for a variety of backgrounds to be represented at each table, which was viewed as beneficial to the discussions.

It was clear, though, that there is a happy medium for the number of participants that provides both diversity of experience and sufficient time for all participants to contribute to fruitful discussion.

> *I think it was fine. I wouldn't put more. But less is maybe too… you may not achieve what you want. I think you need the diversity, but not too many people. (R2a)*

Due to the size and diversity of the groups in terms of experiences, both of cancer and its treatment, and in giving this kind of feedback to researchers, one researcher felt that time was spent at the start in order for everyone to '*get on the same page*' *(R4a)*. However, researchers and patients both recognised that there was a lack of diversity and representation of some demographic groups, because members of the NCRI Consumer Forum inevitably represent a sub-set of the population, and perhaps especially as the majority those who attended had been members for at least two years (supplementary file Appendix D, Box 4). NCRI Consumers are recruited in open competition, mainly to serve on academic research committees or other research groups working at a strategic level, and a successful applicant is required to have some prior knowledge or experience of cancer research.[20]

Facilitation of discussion

The researcher presenting the topic at each table facilitated the discussions. Feedback from the patients and other researchers on how that role was performed was generally positive, but it was suggested that combining facilitation with explaining the research ideas and the main goals of the session was too much, and that an 'independent' facilitator might be helpful. There was also a recommendation that it might be an advantage to have more senior moderators, who would feel more comfortable being directive in keeping the discussions on topic.

> *I think if maybe it had been a more senior person there…who is almost monitoring, keeping an eye on what's going on, giving a helping hand if somebody looks a little bit adrift, they could intervene at that point and get things back on track for them. (C5)*

One point identified for aiding facilitation was a suggestion that more structure to the questions might have helped direct the discussions. Clarifying at the outset what the main point is, where contributions would help, and which issues were in fact less important, may have made the session more fruitful.

Given that the researcher at each table was both presenting the research and facilitating the discussions, having a separate note-taker was appreciated. Another suggestion was to encourage others to make notes, and that the inclusion of visual aids, such as whiteboards or flip charts, might help to clarify the main issues, and focus and direct the discussions (supplementary file Appendix D, Box 5).

For peer review only - http://bmjopen.bmj.com/site/about/guidelines.xhtml

Layout and timing

Layout of the room

The impact of the layout of the room featured for both patients and researchers, who felt that the room was too small and the tables too close together. There was some concern that those with hearing difficulties were disadvantaged by the layout, and the patients, predominantly, felt that the discussions would have been better had the layout had been different.

> *…there were loads of tables in the one room. It was actually quite difficult to hear so that was frustrating in some ways (C2)*

Some also made recommendations about having each group in a separate room, although others felt the 'buzz' of the room helped create a good atmosphere (supplementary file Appendix D, Box 6).

Consideration of special needs

In their feedback, the patients gave some useful comments regarding consideration of the special needs of their group. In addition to the room layout impacting those with hearing impairments (as above), it was suggested that materials should have been provided for note-taking.

> *Everyone having pen and paper to write things down, but having our own space to write things, a turn to speak and then write stuff down to be collected that might help (C1)*

They also suggested reducing the intensity and length of the day, especially considering the long-term health conditions of some of the patients, some of whom were still undergoing treatment (supplementary file Appendix D, Box 7).

Length of the session

The session was scheduled after the lunch break for 40 minutes, followed by a 45-minute plenary session. Given the length of time it took for each table to settle and begin the discussions, and after a few minutes of introduction and background to the topic by the researcher, there were roughly 30 minutes for open dialogue.

Many comments indicated that the time was too short, based partly on the numbers of patients at each table, but mainly on the complexity of the topics being presented.

> *…in trying to put those objectives of my project in the most specific way and in lay terms, understandable way, I think by the time we bridge these initial difficulties we run out of time (R4a)*

In a few groups it seemed that the discussions only made progress towards the end of the time allocated. Both patients and researchers commented that a substantial part of the time was taken at the start of the session to develop a good understanding of the project and where the researchers needed insight from the patients (supplementary file Appendix D, Box 8).

Summary of post-event evaluation by patients

Two-thirds (64%, 29/45) of patients responded to the request for evaluation immediately following the event. The Dragons' Den session was rated as the most informative session of the day by patients. In fact, 86% rated the group session informative for their "personal development as a consumer in cancer research" at some level, and almost three-quarters of attendees (72%) found the session useful for their role as an NCRI Consumer to some degree (Appendix B).

Overall, hosting of each table was overall considered effective ("very effective": 9/29, 31.0%), but just under half of respondents found it only "slightly effective" (13/29, 45%), with a few patients rating it

For peer review only - http://bmjopen.bmj.com/site/about/guidelines.xhtml

neutral or ineffective, or exceptional. Most respondents found the event well organised (93%) and the venue location good (90%), but fewer found the room comfortable (59%).

Recommendations for the future

The lessons learned from this experience have helped inform the Cancer Survival Group's interactions with patients. We hope they will offer guidance to other researchers who want to arrange similar sessions. Although the epidemiological and methodological research in the Cancer Survival Group differs from that on which patients are more used to being consulted (e.g. designing clinical trials), the experience can be fruitful for both patients and researchers.

Suggestions for running a similar patient involvement session

Before the event:

Send detailed "pre-work" which has been piloted with one or two patients first, ideally co-written by them

Explain each study succinctly. Outline the specific questions that the researchers wish to explore. Encourage patients to send questions in advance, to show the researchers what they might want to discuss or have clarified.

On the day:

Ensure enough space between tables so that all can be heard

Ensure the layout is appropriate for the size of groups. Seating arrangements will be dependent on the available space, but ideally, the tables should be sufficiently well spaced that discussions at neighbouring tables are not a distraction.

While some groups may find background silence daunting and the buzz of conversation may help stimulate people to speak or make them feel more relaxed, for this group of experienced patients, most of whom were ready and willing to talk, a layout with more space would have been preferable. In another study that used a similar format of patient engagement, Hill et al[3] found that they successfully dealt with initial issues of noise by spacing the tables out at future events.

We would suggest only two to three groups (tables) per room if possible, so they can be spread as widely as possible. This allows a sense of privacy and ensures that everyone at each table can hear and be heard.

We would recommend excluding those who are not directly involved in the session from the room (e.g. organisers, observers), as we found that these people add to the level of noise and can discourage openness.

Provide note-taking facilities and encourage their use

It is useful to provide pen and paper or post-it notes, and encourage notes to be written down that can be shared with the researchers during and after the session.

A white-board or flip-chart for each group would enable diagrams and technical terms to be written up, so that suggestions can be recorded for all to see. It is a good idea to photograph the boards or flip-charts at the end of the session, before they are taken down.

Allow plenty of time

For peer review only

If possible, take a whole day. Rather than having a one-off session, the day could be split into three discrete sessions, with breaks in between. Time can then be spent first on introductions, as well as allowing patients time to introduce themselves and share their experiences. These all add to researchers' understanding, even if they are not directly relevant to the questions that ultimately need to be answered about each project.

Secondly, allow a generous amount of time for the introduction and initial discussion of the topic. This session can focus on general questions the patients might have to clarify the work, or arising from their 'pre-work'. It should end with the researcher laying out the specific questions on which they would like to focus in the final discussion.

Lastly, run the Dragons' Den session, which should then focus on eliciting specific advice for the researchers to solve the problems they may have been having with study design, or on how patients' experiences could help improve their research question.

Have an independent facilitator for each group

While this type of activity is suitable for all researchers, no matter their seniority, career stage or experience of PPI, we recommend having an 'independent' facilitator for each group who is not directly involved in the research. This should be a senior researcher with experience of chairing meetings who can be diplomatic but firm, while keeping the discussions focused, ensuring everyone is encouraged to speak, and summarising the main points to check for clarity.[3]

After the event:

Follow-up with patients

As well as the usual evaluation forms, we recommend that researchers send follow-up notes to participants so that they can add further comments after having had a chance to reflect. This will add to the richness of experience gained from the day, and use the patients' knowledge to the fullest extent.

It is also important to inform the people who have contributed to the research about what has happened as a result of their input and to let them know how the research has changed or developed as a result. This may be in the form of asking them to comment on research proposals based on the project discussed or in a brief report on its progress.

Remaining challenges:

Several challenges remain. The composition of patient groups was not fully representative of the cancer patient population. This was also noted in a systematic review of patient and public involvement in cancer research,[10] which found that PPI participants tend "to be well-educated female participants from ethnic majority groups" (p.14). Harder-to-reach groups should be invited, if possible, including more disadvantaged patients or their carers, those of different ethnicities and those with language barriers, or their representatives. By definition, it is difficult to include these groups, but efforts to approach them should be considered. For example, if a session were focused on specific cancers (or other disease areas) one or two charities focused on those conditions might be asked to invite participants.

We would also like to be able to include patients with more special needs. Consideration should be given to the support such patients would need, and how it can best be provided.

For peer review only

The Cancer Survival Group is fortunate to have access to numerous teaching rooms, but space was still an issue. In some locations, space may be even more limited. We would still suggest attempting a Dragons' Den event, but perhaps for one or two groups at a time.

Conclusion

We have shared the lessons from one research group's experience of engaging with patients and their carers, in the hope of encouraging other researchers to carry out this kind of consultation, while starting from a more informed position.

The Dragons' Den process is extremely valuable to researchers engaged in all kinds of research, including epidemiological research based on routinely collected data. As well as receiving constructive advice on current research projects, it was also a chance for us to reconnect with the reason we do this research.

From the patients' perspective, participants were keen to contribute to work that would inform research and change policy. They emphasise the need for action and for everyone to "raise their voices" to work for easier access to data for epidemiological research, and to ensure that patients remain at the heart of all the research we do.

This event was held well before the COVID-19 pandemic. For now, the way we run events and activities must change, especially if those events would include people in vulnerable groups e.g. those undergoing treatment for cancer. We believe that very successful patient involvement sessions can still be run using online platforms, using many of the suggestions we have made above.

Similar considerations of pre-work, group size and composition, facilitation, note-taking and timing will be useful in planning online events, and some will come to the fore even more. For instance, we believe that an experienced, independent facilitator will be particularly important in this setting.

Although online events can feel very different, we would argue that most of the same considerations are vital for researchers hoping to run such an event. Having to work remotely should not prevent us from engaging with and involving the patients and future patients whom we hope will benefit from our research.

For peer review only

Figure legends/captions

Figure 1: In the "Dragons' Den". Credit: Photograph by Simon Callaghan Photography

Figure 2: The Dragons' Den session under way, showing the layout of the room used. Credit: Photograph by Simon Callaghan Photography

References

1. Cancer Survival Group. About us: we work with people who have had cancer 2019 [updated 04/07/2019]. Available from: https://csg.lshtm.ac.uk/what-we-do/#cancer accessed 10/08/2020.

2. Hacker KE, Smith AB. Engaging Stakeholders and Patient Partners. *Surg Oncol Clin N Am* 2018;27(4):665-73. doi: 10.1016/j.soc.2018.05.007

3. Hill K, Portman M, Tabi Z. Meet the researchers: an alternative method of engaging patients with research in mesothelioma. *Res Involv Engagem* 2018;4(1):33. doi: 10.1186/s40900-018-0119-x

4. Piil K, Jarden M. Patient involvement in research priorities (PIRE): a study protocol. *BMJ Open* 2016;6(5):e010615. doi: 10.1136/bmjopen-2015-010615

5. Lee DJ, Avulova S, Conwill R, et al. Patient engagement in the design and execution of urologic oncology research. *Urol Oncol* 2017;35(9):552-58. doi: 10.1016/j.urolonc.2017.07.002

6. Pattison N, Lee M. Two tribes coming together: patient and public involvement in cancer research. *Eur J Cancer Care (Engl)* 2014;23(1):1-2. doi: 10.1111/ecc.12178

7. Stewart D, Wilson R, Selby P, et al. Patient and public involvement. *Ann Oncol* 2011;22 Suppl 7:vii54-vii56. doi: 10.1093/annonc/mdr427

8. Gordon L, Dickinson A, Offredy M, et al. A research note on the benefit of patient and public involvement in research: The experience of prostate cancer patients regarding information in radiotherapy. *Radiography (London, England : 1995)* 2017;23(2):167-70. doi: 10.1016/j.radi.2017.02.004

9. Ardron D, Kendall M. Patient and public involvement in health research: what is it, and why is it so important? *Int J Palliat Nurs* 2010;16(4):160-2. doi: 10.12968/ijpn.2010.16.4.47778

10. Pii KH, Schou LH, Piil K, et al. Current trends in patient and public involvement in cancer research: A systematic review. *Health expectations : an international journal of public participation in health care and health policy* 2019;22(1):3-20. doi: 10.1111/hex.12841

11. INVOLVE. Briefing notes for researchers: involving the public in NHS, public health and social care research. Eastleigh: INVOLVE, 2012. [Available from: https://www.invo.org.uk/posttypepublication/involve-briefing-notes-for-researchers/ accessed 10/08/2020]

12. INVOLVE. Briefing note two: What is public involvement in research? [Available from: https://www.invo.org.uk/posttyperesource/what-is-public-involvement-in-research/ accessed 10/08/2020]

13. Thompson J, Bissell P, Cooper CL, et al. Exploring the impact of patient and public involvement in a cancer research setting. *Qual Health Res* 2014;24(1):46-54. doi: 10.1177/1049732313514482

14. Howe A, Mathie E, Munday D, et al. Learning to work together - lessons from a reflective analysis of a research project on public involvement. *Res Involv Engagem* 2017;3:1. doi: 10.1186/s40900-016-0051-x

For peer review only

15. Ocloo J, Matthews R. From tokenism to empowerment: progressing patient and public involvement in healthcare improvement. *BMJ Qual Saf* 2016;25(8):626-32. doi: 10.1136/bmjqs-2015-004839

16. Puts MTE, Sattar S, Ghodraty-Jabloo V, et al. Patient engagement in research with older adults with cancer. *J Geriatr Oncol* 2017;8(6):391-96. doi: 10.1016/j.jgo.2017.05.002

17. National Cancer Research Institute (NCRI). A 'Dragon's Den' workshop to improve research through Consumer involvement 2018 [updated 31/01/2018. Available from: https://www.ncri.org.uk/case-study/a-dragons-den-workshop-to-improve-research-through-consumer-involvement/]

18. Green J, Thorogood N. Qualitative Methods for Health Research. 4th ed: Sage Publications Ltd (UK) 2018.

19. Bradshaw C, Atkinson S, Doody O. Employing a Qualitative Description Approach in Health Care Research. *Glob Qual Nurs Res* 2017;4:1-8. doi: 10.1177/2333393617742282

20. NCRI Consumer Forum. Role Profile for Consumer Members of NCRI Clinical Studies Groups. http://www.ncri.org.uk/wp-content/uploads/2017/02/CSG-Consumer-Role-Profile-PDF-2017.pdf, 2017.

For peer review only

Author Statement

All authors conceived and planned the study. **MM** conducted and transcribed the interviews, gathered and analysed the data. **MM** and **YA** summarised the data, reviewed the literature and wrote the manuscript. **RS, MPC** and **BR** provided comments on the manuscript and all authors have seen and approved the final version of the manuscript for publication.

Data sharing statement

No additional data available.

Acknowledgements

We gratefully acknowledge all the participants of the Dragons' Den session, in particular those who were interviewed after the event. We also acknowledge Dr Edmund Njeru Njagi for his contribution to the collation of feedback after the event and comments on the initial paper outline. Finally, we acknowledge Simon Callaghan who provided the photos from the event and all those who participated and allowed their images to be used. All participants were informed about photography before the event and on the day, and an option to opt out of appearing in any published images was provided and respected.

Conflicts of interests

The authors declare no conflicts of interests.

Funding

YA is supported by Cancer Research UK grant number C7923/A18525

For peer review only

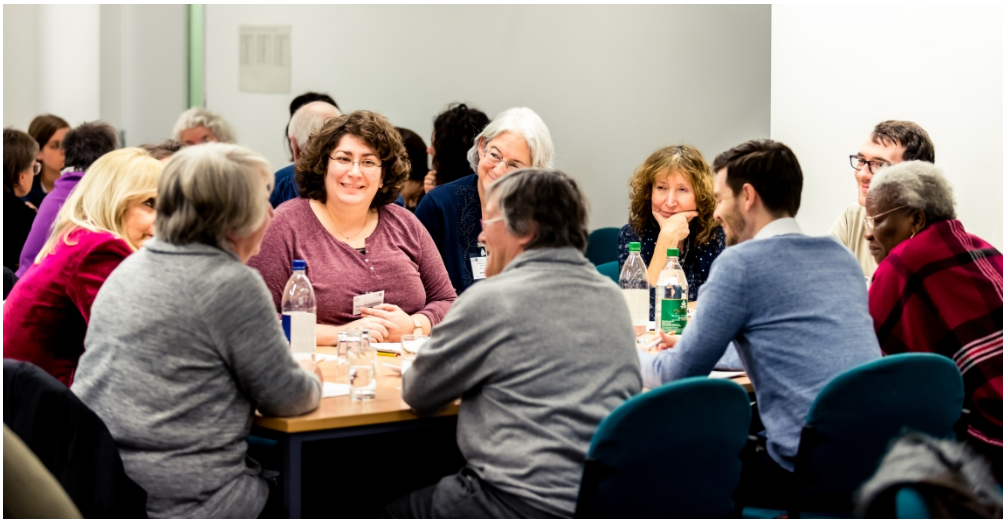

Figure 1: In the “Dragons’ Den”. Credit: Photograph by Simon Callaghan Photography

342x176mm (200 x 200 DPI)

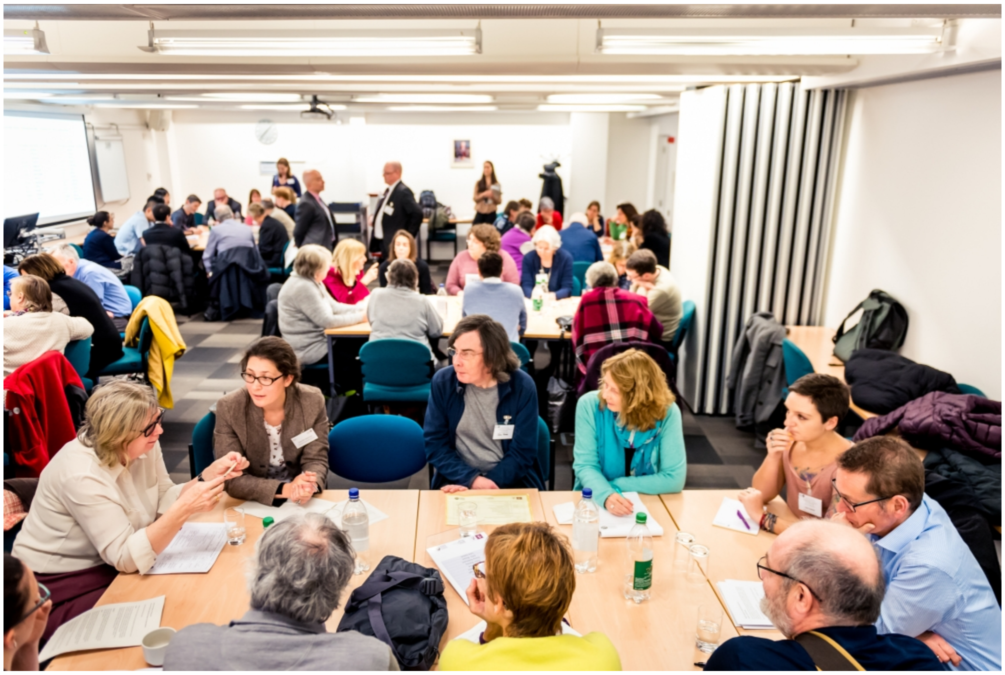

Figure 2: The Dragons' Den session under way, showing the layout of the room used. Credit: Photograph by Simon Callaghan Photography

342x228mm (200 x 200 DPI)

Appendix A: Topics of Dragon's Den tables (Feb 2017)

#	Table Topic	Research summary
1	Opportunities for reducing emergency cancer diagnosis: the role of comorbidities in influencing help-seeking for cancer symptoms and access to diagnostic investigations	We aim to examine the clinical events during the months before a cancer diagnosis in order to identify opportunities to diagnose cancer earlier and reduce emergency cancer diagnoses. In particular, we want to better understand why patients with a long-standing health problem (comorbidity) often have a higher risk of delayed cancer diagnosis and emergency presentation.
2	Examining inequalities in cancer survival for colon cancer patients living in London and treated with surgery in a London hospital	I am looking into identifying the main drivers of these differences in survival including factors such as: CCG of residence, hospital of surgery, socio-economic background, stage at diagnosis, specialised colorectal-surgeon, discussion of treatment plan in a MDT.
3	Evaluation of public health interventions	We aim to evaluate public health cancer policies soon after their implementation or even before their implementation. Using the technique of 'scenario modelling', by which we artificially change the composition of our cohorts of patients and how they are managed, to reflect the likely impact of a policy, we would like to look at the effect of those policies on cancer survival and other derived measures.
4	Factors influencing decision-making in cancer care.	These studies aim to explore which factors influence doctors' and patients' decision-making regarding the management of an aggressive type of Non-Hodgkin Lymphoma called Diffuse Large B-Cell Lymphoma (DLBCL) and/or bowel cancer. In particular, we are interested in people's attitude towards clinicians' management of elderly cancer patients.

For peer review only

**5**	Inequalities in cancer management and survival: NHS processes and/or lack of resources?	Some groups of cancer patients, for example from the more deprived categories, experienced lower survival from their cancer. It has been shown that these patients do not receive the most optimal management (including diagnostic investigation and treatment), and their higher proportion of comorbidity and more advanced stage at diagnosis would explain only part of these inequalities in cancer management. The reasons these patients are, for example, more likely to be managed in non-specialised hospitals or to be operated by less experienced surgeons are not understood. We aim to investigate the role of the organisation (processes) of the NHS and its disparities in resources, in these inequalities in cancer management and cancer survival.

For peer review only

Appendix B: Evaluation of relevant parts of the day given within a week of the event

	No new information	Reinforced existing information	Slightly informative	Informative	Extremely informative
Group session	0 (0.0%)	4 (13.8%)	8 (27.6%)	4 (13.8%)	13 (44.8%)
Plenary feedback session	0 (0.0%)	4 (13.8%)	6 (20.7%)	6 (20.7%)	13 (44.8%)

	Topic not relevant to me	Relevant but not useful	Slightly useful	Useful	Extremely Useful
Group session	1 (3.4%)	6 (20.7%)	5 (17.2%)	6 (20.7%)	10 (34.5%)
Plenary feedback session	1 (3.4%)	4 (13.8%)	7 (24.1%)	7 (24.1%)	9 (31.0%)

	Ineffective	Neither effective nor ineffective	Slightly effective	Very effective	Exceptional
Table hosting	2 (6.9%)	1 (3.4%)	13 (44.8%)	9 (31.0%)	4 (13.8%)

	Unsatisfactory	Neither satisfactory or unsatisfactory	Slightly Satisfactory	Very satisfactory	Exceptional
Event organisation	0 (0.0%)	1 (3.4%)	1 (3.4%)	21 (72.4%)	6 (20.7%)
Building and location	0 (0.0%)	0 (0.0%)	2 (6.9%)	20 (69.0%)	6 (20.7%)
Room and comfort	1 (3.4%)	2 (6.9%)	9 (31.0%)	12 (41.4%)	5 (17.2%)

For peer review only

Appendix C: Semi-structured interview questions

Interview questions for consumers

1. What were you hoping to gain from participating in the Dragon's Den session?

2. Did it meet your expectations?

3. Do you feel your comments were listened to and taken on board?
    a. How did the researcher make that possible?

    OR b. What was it about the session that meant that didn't happen?

4. Did you feel that the feedback provided by the consumers will make a positive difference to the project?

5. Did you feel that there was enough opportunity for questions and discussion?

6. Was the session the right length/layout/number of participants?

7. Do you have any comments or suggestions for improvements that we could make to the session in order to better meet the needs of researchers and/or consumers?

8. Any other comments?

Interview questions for researchers

1. What were you hoping to gain from presenting at the Dragon's Den session?

2. Were you able to achieve those objectives?

3. Did the session meet your expectations?

4. Do you think the feedback provided by the consumers will make a positive difference to the project?
    a. In what way?
    b. Or why not?

5. What do you think the consumers got out of the session?

6. Did you feel that there was enough opportunity for questions and discussion?

7. Was the session the right length/layout/number of participants?

8. Do you have any comments or suggestions for improvements that we could make to the session in order to better meet the needs of researchers and/or consumers?

9. Any other comments?

For peer review only

Appendix D

Box 1

Overall experience (what did you get out of the session?)

It's an opportunity to hear what other people think about something, expanding my own learning and it gives me more experience that I can draw on in the future…So for me it's about trying to get my bit across, listen to everyone else, and try to learn a bit more, it's about a learning process (C2)

All the topics were relevant, it was well planned, relevant to patients with cancer, living with and beyond cancer... There is a feeling of altruism wanting to give back and help someone's knowledge and experience so perhaps this information will help others being treated in the future…I would like to see it repeated because I think it was a very fruitful event. (C3)

I enjoyed it very much and got a lot out of it. (C4)

I was thrilled at that opportunity to discuss with Consumers...it was a great experience, and I wish we had more opportunities to share about the work we do in that context (R1a)

I thought it was a very useful exercise and patient representatives were really active and each of them really had something to say on the topic. So, I think it was a really useful interaction with patients. (R3a)

It was the first time I was involved in the Dragons' Den, I would like to definitely to repeat it (R4a)

I was excited to get some information from people with first-hand experience of cancer. (R4b)

On the actual day, the sessions were largely as I had expected them to be. The participants were very…keen to share their views and experiences, which were very insightful. (R5b)

It was a lot more difficult than I thought. I didn't really know what we were there for, so it was a bit challenging at times. (C1)

I was also aware that there may not be easy answers to our research question. The session was interesting but also quite difficult. (R4b)

Because it went so well actually I wanted to go beyond to have more information, to discuss more etc, and that was quite frustrating. And for everyone I think. (R2a)

I was a bit stressed, to start with, because I had no clue…if I would have to do the talking non-stop or the listening non-stop, and I think it went well overall, but it kind of got out of control…so in the end, I was happy when it finished, it was enough. (R1a)

Box 2

Expectations / Importance of patient contribution

Working together towards improving outcomes for patients is the best way to achieve reliable results… the genuine information can come from those who have experienced the situation undergoing that, dealing with things…As a colleague on an equal basis everyone working side-by-side to discuss the research and receiving equal consideration it's so important I think to provide this opportunity. (C3)

I believe the answers provided by the Consumers will provide more clarity on the question and the practicalities to be considered in the research. (C4)

You don't always know whether you are really being listened to or not because people are polite generally speaking, so therefore they sometimes can maybe appear to be interested. You don't know when they go away, whether they actually do anything with it or not. But I was aware in this case that they did, so that was very positive…I mean the researchers have a very good idea of the overview of what they want to get to, but sometimes it's the practical issues that the PPI [Public and Patient Involvement] can throw in. (C5)

It obviously did turn out to be pertinent. They showed that by their response at the time and I thought that those issues were incorporated in the grant proposal that we got to read later on...So, it was actually made very obvious that they did take that on board… (C5)

For peer review only

I don't know that I've heard very much what about what will actually happen, what the next steps are…it might be useful if there's something you could pass around at the end of the session to say "This is where this particular project is, here's the written summary of what you said" just some way of feeding that back. It was all said verbally. (C2)

So, I was hoping to get somehow, an insider's view of what matters to the patients really in terms of, or what they think could improve survival, somehow, stuff that could be addressed by policy makers. (R1a)

I think that it's useful to have the views from the patients to know that you're on the right track…And I hope they see that we value their point of view and their opinion and their contribution and how we have the need to hear what they think about the issues we are trying to study. (R3a)

The patients were well informed; their answers were precise, informative, and not just anecdotal. (R2b)

It brings a different perspective and different insights. (R4a)

It was touching to listening to their experiences and I appreciated the fact that most people were open about their personal experiences. (R1b)

Like all these things, they seem very obvious to them, but of course you won't get that sense from looking at registry data... It was a good reminder for us that we sit in front of the computer and it loses that personal touch…it felt really intense to hear their experience. (R5a)

Box 3

Content of discussion

There were a lot of us that for a while didn't really understand what was being asked of us and how we could help with the research. We did eventually but there was quite a while where we weren't quite sure for a while what exactly we were supposed to be inputting…it took us a while to figure out what the point to it was exactly. That wasn't clear at all, it was the nature of the study I guess (C1)

I think at the beginning of the session, it wasn't as clear as it could be exactly what the researcher was wanting to ask us. (C4)

There were sort of two bits to it, so it would have been easier if there had only been one piece rather than two angles. (C5)

There were a few steps that the Consumers needed to understand before they could really answer things that would be useful to me…So all of these concepts were quite tricky to grasp, and I discovered it as we were talking with the patients as well…It's more of a stats [project] so for me it was hard to translate into words and applications, and for them it was hard to get the stats concepts behind…I would have picked another subject I guess. (R1a)

I presented a very broad project…but I think it was too broad. (R4a)

But after a bit of fumbling we were definitely able to come up with a lot of stuff (C1)

We didn't have enough time to discuss: "So what? What can we do etc?" What I wanted to discuss, "Now there are some initiatives to try tackle that, or maybe not, we don't know - what do you think? Do you think it worsens the situation or does it help?" And we didn't have time and I think it would have been very useful. (R2a)

Box 4

Composition of groups

The number of participants, I think it was a good number and it was useful to have to people from different experiences, ages, and carers and patients and so I think it was a good mix and the number was right... And the fact that they were men and women, different age groups, I think that was very interesting, carers and patient[s]…If there was a bit more variety in terms of background of the participants, it would maybe make the discussion even richer, even open up new insights…The only downside I think was that these were all people who were highly engaged, eloquent, had very clear views, had thought about it a lot. So, when we

very briefly talked about the issue of lower SES, obviously there wasn't anybody there representing those groups so that was a bit of a limitation. (R3a)

I thought the number of participants was good, I thought the breadth of experience of the participants was good… In terms of representation, that was very good (C4)

I think it was fine. I wouldn't put more. But less is maybe too - you may not achieve what you want. I think you need the diversity, but not too many people. (R2a)

I think less people, not many less people but a few less people around the table would have been good, especially for the length of time… (C1)

…we are not representative of the population. It's the same with most patient reps - it tends to be…well educated white middle class with sharp elbows…they have resources in terms of time and education, can put some time aside and can make a difference, but we're not necessarily reflecting the population that we should reflect, given that cancer is a disease, certain cancers are more prevalent within certain communities. (C2)

I went away with the feeling that this is such a selected group … they are active patients because they're involved in these sort of things... Because they are there, and they are patients who they know that they can be useful for research (R5a)

Box 5

Facilitation of discussion

I thought [the researcher] was an excellent facilitator. Fortunately, Consumers are very talkative, so it's quite a job to not just comment and keep things going but try and keep things within the thing that you're supposed to be talking, trying to keep things moving along and I think that was done well (C2)

Yes – it was an open discussion, and everyone at the table could contribute and those that weren't able to easily contribute for example, having hearing difficulties, everyone was made to feel that had the same input as others, in a position where they were able to contribute appropriately…, I do remember the researcher being engaged with everybody. (C4)

[The lead researcher] did a good job in describing the topic and engaging members and handled the questions well by triggering different conversations (R1b)

I think it was tricky as the presenter to be presenting my topic and also moderating the discussion because I don't have those skills, of a moderator, and I also had to get my point across and I had to welcome everyone's suggestions so that everyone would feel free to discuss and that was quite tricky…It was really good that I didn't have to take the notes, because I think that would have been really hard… I made sure I was engaged in making eye contact. (R1a)

I think if maybe it had been a more senior person there … who is almost monitoring keeping an eye on what's going on, giving a helping hand if somebody looks a little bit adrift, they could intervene at that point and get things back on track for them… if you can use a whiteboard or a flipchart, not to make it too long, but a picture always can speak a thousand words and if you can draw a little diagram or a couple of words that sound very difficult, write them up so that people are clear what you're talking about. It makes it a little bit easier both for them to explain and for the other people to understand (C5)

[A consumer] was the one that actually tried to somehow focus the others, saying, "No, wait a minute, we're here to discuss this and this," because obviously [we were] not reaching that common point of understanding for all…I think the key is the structuring a priori because that would avoid … cross talking because it's natural, it's open and that's what happens (R4a).

Box 6

Layout of the room

The acoustics weren't very good, and the layout of the table was such that sometimes it was hard to hear what was being said by maybe another discussant at the far end...Either the room needed to be a little bit

For peer review only

bigger, so that the tables were more spaced out, which is what we would generally have. Or needed to be in a separate little sub room where you could talk a little bit more readily. The main thing I felt really was that the room that was used wasn't terribly great, it was squashed and too noisy and that caused issues (C5)

…maybe separate rooms with complete silence around would have helped a little bit. (R1a)

…there were loads of tables in the one room. It was actually quite difficult to hear so that was frustrating in some ways because on that small table if there had been more space a lot of us would have got a lot more out of that particular meeting (C2)

…it's always that thing of hearing what's being said at other tables, adjacent tables, but that's really environmental and beyond the control of the school itself. I do wonder if other participants…might feel a little hesitant about expressing, describing their personal situation…(C3)

For me I'm a little deaf, then it was hard because we had a lot of tables, it was a little noisy…we would need probably more space because the acoustics are not really good…You know, in a way it's nice to see this, I think it's - you know you are part of a big event in a way. It can be quite nice. While if you are all in different rooms… (R2a)

The room was loud, and a participant complained they could not hear or contribute to our discussion. (R4b)

Box 7

Consideration of special needs

There were a couple of people at our table who were a little older. And because [of that] they weren't as quick to pick up in terms of hearing, and they found the noisy background a bit of a distraction at times. (C5)

I think the noise could be a problem for anyone with hearing difficulties…there was always a low buzz of conversation which is always natural when people are speaking (C3)

…because there were a couple of people at the tables who had hearing issues, and they did find it hard to hear, so that could have been done better. (C4)

It was quite a full-on day, all day long and I don't think anybody can say that they were 100% engaged 100% of the time… (C2)

Everyone having pen and paper to write things down, but having our own space to write things, a turn to speak and then write stuff down to be collected that might help (C1)

Box 8

Length of the session

I think the session was also too short for all the patients and carers to make a full contribution to a complex topic. (R4b)

…a little bit limited at the end because time was running out but again I don't think that was anyone's fault, it was just that that the topics were so interesting so that many people wished to contribute. I think in a way that's a sign of a very successful event. (C3)

…I tried to include everyone, but it was very difficult…(R5a)

…in order for everybody to be able to contribute to the discussion we had to really move forward quite quickly and so everybody could maybe say a few sentences and then we couldn't go really in depth. So, it was more like the start of a conversation… If we had slightly more time it would have to be useful for going a bit more in-depth. (R3a)

…to me it was really ending quite quickly, when we were really getting into it better as we got towards the end…I thought the discussion was getting much more animated as time went on. At the beginning it took a little bit of time to present what they were doing. (C5)

For peer review only

Because I feel that the time we had until we really got into the details of the projects, we ran out of time for that reason… maybe in the last three or four minutes, we reached basically the beginning of the discussion I wanted to have. Also, from my perspective in trying to put those objectives of my project in the most specific way and in lay terms, understandable way, I think by the time we bridge these initial difficulties we run out of time (R4a)

Yes, we were told to stop, so we could have gone for longer. Maybe part of it was that it took us a while to figure out what was going on… (C1)

For peer review only
